# The Multi-Player Performance-Enhancing Drug Game

**DOI:** 10.1371/journal.pone.0063306

**Published:** 2013-05-17

**Authors:** Kjetil K. Haugen, Tamás Nepusz, Andrea Petróczi

**Affiliations:** 1 Faculty of Economics, Informatics and Social Science, Molde Univeristy College, Molde, Norway; 2 Department of Biological Physics, Eötvös Loránd University, Budapest, Hungary; 3 Faculty of Science, Engineering and Computing, Kingston University, Surrey, United Kingdom; Cinvestav-Merida, Mexico

## Abstract

This paper extends classical work on economics of doping into a multi-player game setting. Apart from being among the first papers formally formulating and analysing a multi-player doping situation, we find interesting results related to different types of Nash-equilibria (NE). Based mainly on analytic results, we claim at least two different NE structures linked to the choice of prize functions. Linear prize functions provide NEs characterised by either everyone or nobody taking drugs, while non-linear prize functions lead to qualitatively different NEs with significantly more complex predictive characteristics.

## Introduction

Doping in competitive sport is a peculiar phenomenon. The need for performance enhancement is emerging from the desire to maximise or even expand human capacities [1], and by doping to gain competitive edge in a situation where athletes’ performances are judged on two levels simultaneously: athletes compete against the opponents in situations where typically only one can win and are also automatically entered into a quest for breaking records which opens up the competitive arena including all from the past. From the array of substances with performance enhancing properties, a wide range represents fully acceptable means, whilst a defined set is prohibited by some authorities. In general terms, and for the purpose of this paper, the term “doping” refers to the latter category.

From the system’s point of view, the current detection-based anti-doping policy does not automatically eradicate the use of prohibited substances, but rather presents a barrier with a quantifiable risk of being caught. It is easy to see that such a system leads to two primary strategies employed by the athletes: i) compliance, driven by respect for the rules, desire to compete clean or fear of being caught and ii) circumvention, i.e. outwitting the system by using not-yet-known or undetectable substances, masking or simply betting on chances of not being selected for testing. Just before the creation of the World Anti-Doping Agency, then-International Olympic Committee president Juan Antonio Samaranch voiced his opinion that doping should be acknowledged and allowed as long as it is safe [2]. The fierce reaction by the public and sporting community has led to tightened rules, creation of the World Anti-Doping Agency (WADA) and an ever increasing demand for investment into testing [3]. We will show that a situation where all athletes end up using doping is possible even under the current prohibiting-detecting anti-doping system, putting their health further in risk by using drugs and masking the use at the same time, increasing the drug intake and the possibilities of side effects from individual drugs and drug interactions.

In order to design effective anti-doping measures, gaining insight into the driving forces behind doping behaviour is vital. Whilst the doping decision is very complex involving moral, economical and health considerations, theoretically this complexity can be distilled into a simple decision situation where pros and cons are weighed against each other in the context of unknown but assumed choices of the opponents. Based on the assumption that eradicating doping from sport requires a significant change in this decisional balance; by formulating and formally solving multi-player doping games, we aim to make a contribution to developing a better understanding of doping decisions.

In this paper, first we review the literature pertaining to the game theoretical approach to sport and in particular, doping. We set up a model for a doping game involving more than one pair of opponents. This extends the classic doping game to a multi-player situation, which resembles many actual doping situations better than a game restricted to two players. We then report analytical solutions for 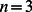 and 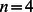 cases complemented with two important propositions. Finally, we discuss both theoretical and practical implications of the multi-player extension of the doping game and offers directions for future research.

## Relevant Literature on Economics of Doping

For athletes entering a high level sport competition, it is a requirement to abide by the rules as set by the relevant governing body. Philosophers would argue that at this point, the question whether the athlete ought to follow these rules or whether they have a reason to do so is contested and depending on the outcome, a decision is made about the action which follows. With regard to doping, athletes must decide whether they feel that they are under obligation (normative) or have a compelling reason (rationality) to obey the rules and refrain from using a prohibited performance enhancing substance.

### Homo Economicus: Pay-offs and Sanctions

Lay explanation for doping rests on the assumption that for those who engage in doping practices “winning is everything”, hence they use prohibited methods to ensure this outcome. This approach assumes that the choice is purely rational-economical and responds to externally imposed incentives and deterrents [4]. As long as the perceived advantages from using doping constitute a far better scenario than any scenario with no doping, factoring in the risk of being detected and its consequences, the only logical action is to dope.

Contrary to the detection-sanction based deterrence methods, economical models recognise the importance of the prize structure, considering both benefits and costs. Prize structures can also be manipulated so the monitoring cost is kept low [5]. These models [6]–[12] suggest that eradicating doping would require changes in the external factors, such as increased dis-utility (including the chance of being detected and its consequences), decreased utility (reduced pay-off) or some combination of the two. In reality, it is unlikely that such change will be effectively implemented. Based on a review of economical models of doping, [13] posit that rank order contests such as sport competitions with highly skewed prize structures inevitably lead to undesirable practices (i.e. doping) players may employ in order to enhance their chances to finish in positions with high pay-off. A follow-up study with empirical data from thirteen different athletic events reinforced the assumption that increase in competition increases doping [14], and consequently lead to a quest for sophisticated detection and a requirement for equitable sanctions.

The nature of doping makes policing difficult and leads to an imperfect but costly monitoring system, where externally imposed sanctions may inadvertently motivate doping use by indicating that doping is widespread, hence the need for harsh sanctions [15].

### Game-like Situations

In addition to the mainstream doping research, a distinct direction (economics of doping) evolved around game theoretical approaches, focusing on different combinations of the normative determinants. Early theoretical exploration of the doping dilemma focused on typical game strategies athletes may engage in, starting from 2-player symmetric and asymmetric games [16], [17].

An empirical cross-sectional investigation, using ranked outcomes of best, next best, next worst and worst, concluded that “ruthless”, winning-at-all-cost-type athletes clearly dominated the doping user group but drug users were also found among Naessian-type athletes who value the process more than the outcome [18]. Although very tentatively, a possibility to change games upon some personal experience was also suggested. This evolutionary characteristic is in keeping with Berentsen and Lengwiler’s [19] cyclical dynamics of mostly honest or fraudulent games among heterogeneous players. Subsequent game theoretical models (e.g., [20], [21]) have shown that the likelihood of doping is the function of i) intensity of the competition, ii) the efficiency of the detection system, iii) sanctions if caught, iv) distribution of the prizes and v) health costs.

Although several authors have discussed the obvious extension into a multi-player setting, few formal attempts on modeling or solving multi-player games exist in sports economics research. [8] suggested that athletes with a win-at-all-costs attitude are likely to find doping use the optimal strategy, regardless of the number of other athletes who dope. Expanding on the previous work, [17] also noted that a tendency toward doping use exists in n-player games. Whilst this body of work draw attention to an important element (i.e. sport competition is more often than not is an n-player game), none of these offered analytical or numerical proof.

Berentsen and Lengwiler’s doping evolution game [19] expands the 2-player setting to a multi-player model. However, despite the multi-player setting of heterogeneous (weak and strong) players, decisions are made in pairs. Hence the model avoids explicit multi-player game formulation. The closest attempt to formally investigate multi-player effects is found in Strulik’s model [22]. In order to avoid the complexity of a “real” multi-player formulation, the utility received by player 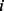 in season 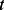 (according to the authors notation) does not depend directly on other players’ actions in season 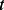. Other players’ actions are built-in into the variables such as the fraction of players taking doping in season 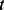 (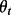).

Considering the existing models to date, sports economic research appears to be lacking formal analysis of multi-player doping games. The aim of this paper is to address this gap by extending the classical doping game theoretical model to a multi-player game.

## Model Set-up

For this model, we consider athletes as those who are in the Registered Testing Pool (RTP) by being identified as elite performer by their respective governing bodies (thus subject to doping testing at any time including sport events) or based on their ranking at the sport events. The RTP includes international level athletes being tested by their international sport federations, and international and national level athletes being tested by national anti doping organisations, or in some cases, national governing bodies of sport, National Olympic Committees and Regional Anti-Doping Organizations. In this respect, there is no distinction between amateur or professional sport. Consequently, we consider prize as the chance of winning vs. the chance of being caught and sanctioned. However, we recognise the fact that in real life, both affects the athlete’s ability to benefitting from sponsorships, endorsement deals and prize money.

### Assumptions and Basic Modeling

We start with the performance-enhancing drug game of [21], also discussed by [23], with the aim of adding multi-player capabilities. We try to stay as close as possible to the original model, only doing minimal necessary enhancements in order to investigate the multi-player case.

We assume the following:

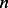
 athletes compete in a certain (one-shot) sports event.
2. Each athlete has a binary choice between taking performance enhancing drugs (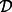) or keeping “clean” (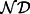).
3. All players are (at least initially) assumed equally good. This “cloning” assumption is merely a direct extension of the original assumption of [21], and it will be discussed in some more detail in the section titled Conclusions and suggestions for further research.
4. Drug testing is routinely performed on all athletes, leading to a certain probability of exposure of 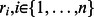, given that drugs have been consumed. Here, we make a slight generalization compared to [21], allowing for different exposure probabilities between athletes. In practice, one could for instance assume either that previous history has trigged different testing programs between different athletes (countries) or simply different doping prevalence between different athletes of groups of athletes (countries).
5. The cost of exposure is again extended similarly as in 4) above to 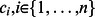. This extended assumption opens up for the model-wise ability to investigate different group specific costs related to drug exposure. It may for instance seem reasonable to assume that certain countries “forgive” dopers easier and earlier than others. Hence, variable 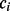
’s may introduce a certain difference in cultural views on performance-enhancing drugs.
6. Drug tests are assumed perfect in the sense that anyone who have not consumed drugs will not be exposed through a test.
7. There is only one drug available, and the effect of the drug is assumed to be equal for all athletes 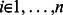. This assumption is also a strict copy of the original assumption, it can of course be relaxed at later stages.
8. The given drug is assumed effective (with certainty) in the sense that if any athlete on drugs compete against any “clean” athlete, the outcome is a certain victory in the sports competition for the drug consumer.

So far, no really important assumptions have been made compared to the original model [21]. We have opened up for athlete specific exposure probabilities 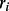 as well as exposure costs 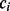. Still, these assumptions are merely direct extensions to the original model.

However, the utility structure of this model needs some careful remodelling. In the original model, a binary type of pay-off structure was defined. In the multi-player case, it seems obvious that a “winner takes all” pay-off structure is neither realistic nor practical. Hence, it seems evident that a more flexible pay-off structure is necessary. Let us furthermore define:

9   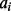 is the utility (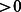) received by athletes through their ranks in the sports        competition. That is, 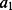 is the utility received by the first prize winner, 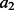 is the        utility received by the runner-up and so forth.
10  Furthermore, it seems reasonable to assume:

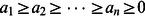
(1)This multi-player model opens up for some different and interesting options regarding the interpretation of solutions. In the original model with 2 players, a concept like doping prevalence was neither very interesting nor very realistic to focus on. In the case of a multi-player game, the prevalence becomes both relevant and tractable.

Let us now formally define decision variables for the players/athletes:
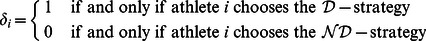
(2)


Given the definition of the binary decision variables 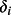, the sum 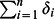 may directly be interpreted as the group prevalence. Hence we define
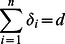
(3)where 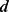 is the prevalence, i.e. the number of athletes choosing to take drugs. With 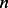 denoting the total number of athletes, 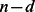 then constitute the number of athletes not taking drugs.

Given the above assumptions 3) and 8), there will be two groups, one group taking drugs and another group keeping clean. The first 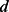 athletes (ranked first, second, 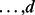
th) take the drug, while the 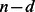 “clean” athletes will occupy ranks 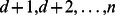. We refer to this two groups as the 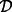-group and the 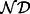-group, respectively. Again, given assumptions 3) and 8), all drug-takers are equally good and all “clean” athletes are equally bad, leading to the following probability densities for the two groups:
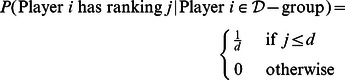
(4)

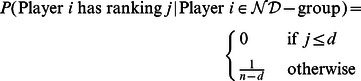
(5)


### Pay-off Functions

Now, given assumptions 1) - 10) as well as derived equations (4) and (5), the pay-off functions of the players can be defined. It will prove convenient to define pay-off functions for the 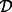-group and the 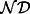-group separately. We define:
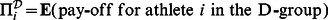
(6)and



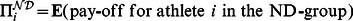
(7)

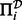
 and 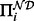 can be expressed using straightforward expected value calculations as follows:
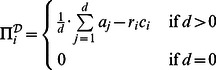
(8)and



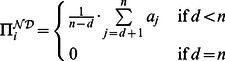
(9)It is straightforward to show by applying equations (8) and (9) that the original 2-player performance-enhancing drug game [21] emerges as a special case of the above model. The content is left for Supporting Information File S1, section 1.

### Analytical Results

We performed a full scale computation (finding all possible NEs) for the three-player case (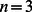) while we settled for a partial analysis for the 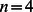-case. The reason is partly due to the overwhelming amount of analytic work involved, but more importantly, our analytic efforts lead us to formulate and prove two propositions which answer quite many relevant questions related to the behaviour of the multi-player performance-enhancing drug game.

Results for the 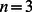-case are shown in Tables 1, 2 and 3. The corresponding derivations and the results for the 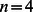 case are included in the Supporting Information File S1.

**Table 1:** Summary of the three-player case - non-linear prize function.

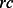	Cases of Nash-equilibria
Less than 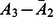	DDD
Exactly 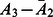	DDD, DDF if 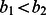 or DD* if 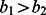
Between 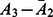 and 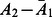	DDF, Dmm, mmm
Exactly 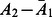	mmm with 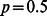
Between 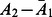 and 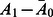	DFF, Fmm, mmm
Exactly 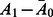	DFF, FFF if 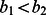 or FF* if 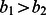
Greater than 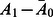	FFF

**Table 2:** Exact probabilities of mixed Nash-equilibria for the three-player case. 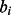 is defined as 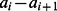, i.e. the prize difference between rank 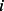 and rank 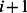.

Case	Probabilities
mmm	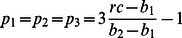
Dmm	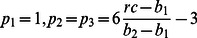
Fmm	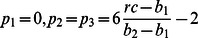
DD*	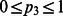 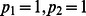 ;
FF*	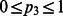 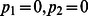 ;

Note that DD* and FF* appears only if the prize function is convex, i.e. 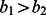.

**Table 3:** Summary of the three-player case - linear prize function.

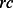	Cases of Nash-equilibria
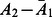 Less than	DDD
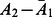 Exactly	***
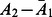 Greater than	FFF

As a simple decoding scheme is used to describe various Nash-equilibria in Tables 1, 2 and 3, some further explanation is necessary. A letter describes the behaviour of one player. Each letter may be D (doping with certainty), F (plays fair with certainty), m (mixed strategy) or * (any pure or mixed strategy). For instance, DDF describes a strategy where two players cheat and the third plays fair all the time, while mmD is a strategy where two players play mixed strategies and the third cheats all the time. The order of letters does not matter, mmD is the same as mDm or Dmm. Note that there is a fine distinction between m and *: m denotes a player playing a mixed strategy with a specific probability of doping, while * denotes a player that can take drugs with any probability without any effect on its own payoff. Furthermore, Tables 1 and 3 contain 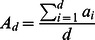, i.e. the average prize received by a player that cheated if there are 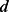 cheaters in total. 
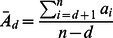
, i.e. the average prize remaining for a fair player if there are 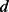 cheaters is also introduced. Note that 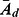 also is a function of 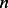 and a more correct notation could be 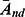. However, to avoid notational confusion, we stick to this simplification.

As can be readily observed from Tables 1 and 3, certain 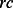-thresholds 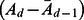 compose various regions of different NEs. Similar 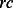-thresholds are found in the four-player case, leading to a “regionalization” that is very similar to the three-player case, but the number of regions is one larger and some of the conditions are more complex to allow for cases when the prize function is not strictly concave or strictly convex (unlike the three-player case, where every prize function is concave, convex or linear). A more comprehensive discussion and derivation of the four-player case is to be found in the Supporting Information File S1.

Comparing Tables 1 and 3 indicate a striking difference. Apart from the (obviously) undetermined 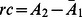 case, either everybody plays fair or everybody cheats if the prize function is linear, while more complicated Nash equilibria may appear if the prize function is concave or convex. The question of whether this simple NE characterization also is present in the 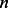-player case led us to formulate (and prove - refer to Supporting Information File S1, section 2 for the proof) the following:

**Theorem 1** Given an instance of the 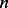-player doping game, a common 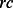 product for all players, and a linear prize structure (i.e. a common 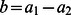), the Nash-equilibria are as follows:

1. Everyone cheats when 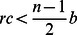.

2. Everyone plays fair when 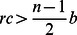.

3. Any pure or mixed strategy when 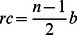.

Possible practical implications of Theorem 1 are discussed in the Conclusions section.

One obvious point to look at when analysing a multi-player version of the performance-enhancing drug game is how NEs evolve as the number of players change. Furthermore, as predictability (normally) is viewed as important related to doping regulation, general knowledge related to when (under what parametric conditions) will NEs be pure or mixed might prove valuable in a practical context. These questions (and others) are to some extent answered by the following theorem (refer to Supporting Information File S1, section 2):

**Theorem 2** Given an instance of the 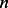-player doping game and a common 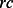 product for all players, the sufficient and necessary condition for the “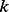 players cheat and 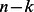 players play fair” pure strategy to be a Nash-equilibrium is as follows:
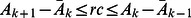
where 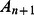 and 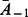 are defined to be 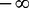.

As mentioned above, one obvious thing to look at when analysing a multi-player game, and comparing it to a two-player version, is how NEs change when the number of players change. Theorem 2 may be applied more or less directly to shed some interesting light on this question. Let us define 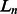 as the length of the interval 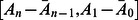; that is,
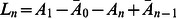
(10)


This interval contains NEs which are either mixed or pure with a mixture of player behaviour. Hence, outside this interval either everybody plays fair or everybody cheats. If we can identify the behaviour of 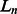 as 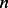 increases it could be meaningful in a regulative setting. Let us investigate the contents of 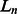 closer.


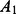
 is by definition the maximal prize 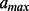, while 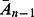 is the minimal prize 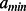. The remaining two elements of 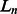 are both equal to the average prize, 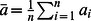. Hence, equation (10) can alternatively be expressed as:
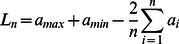
(11)


In order to judge 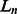
’s monotonicity, it turns out to be easier to assume a normalized prize structure. That is, we convert the given prize function to a version where 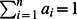. Given this assumption, equation (11) can be further simplified to:
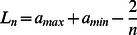
(12)


Assuming that 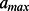 and 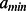 are constants, the only variable in this equation is 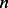. When n increases, 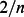 decreases, 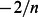 increases, thus 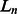 increases. But since 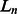 is the length of the unpredictable region with mixed NEs or pure NEs with mixed player behaviours, the length of the predictable region decreases. Therefore, when 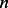 increases, 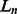 increases, thus the length of the predictable region decreases.

## Conclusions and Suggestions for Further Research

### Conclusions

Based on our results, one obvious conclusion to draw is the extent of complexity increase in NE structures observed simply when moving from a two-player to a three-player game. The contents of Table 1 shows clearly that almost any possible NE can be a game prediction. This constitutes a major difference from existing game theoretic doping research - see for instance [21], [20] or [23]. This is perhaps not surprising as such, but we did not predict such a complexity increase before conducting this research. In any case, some caution should be taken when generalizing doping research based on two-player models when practical regulative means are considered.

**Figure 1:**
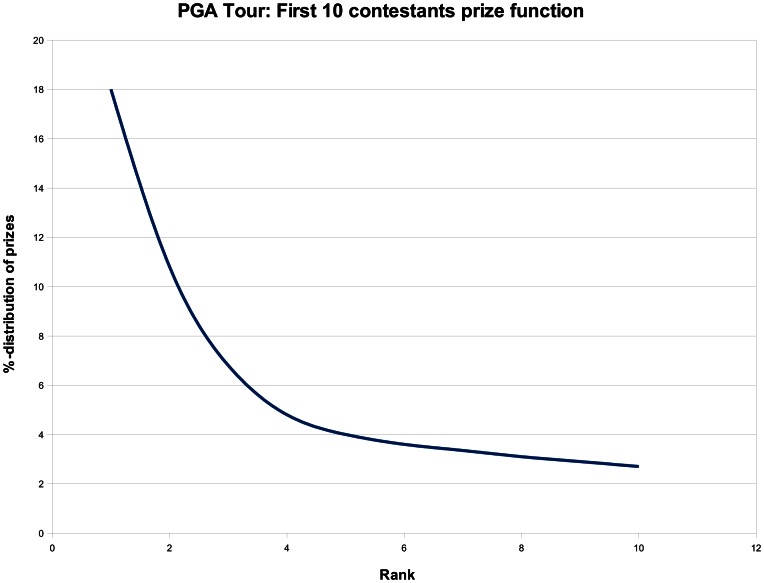
The “prize function” of the PGA tour [28].

Furthermore, the results derived through Theorem 1 provides valuable insight. A popularized version of the theorem could be to say that if the prize function is linear and 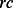 is constant (homogeneous athletes and doping tests), the 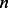-player game behaves just like the two-player game. By itself, this is an interesting result, but we should be cautious in drawing policy implications too far. It could be tempting to say; then it is easy to fight doping, both in 2-player as well as in any-player competitions, by making all drug testing as well as punishment standardized and change all prize functions to be linear. If that can be achieved, then merely a single doping test on one athlete is enough. Surely, this is way too far drawn. As Figure 1 indicates, real world prize functions are not linear, and they should not be linear either - see for instance [24] and [25]. The point is very simple, demand for sport is (typically positively) related to the effort that the athletes put into competing. Fans want to see athletes trying harder, an egalitarian prize function does not lead to optimal performance, and hence, the problem of fighting doping can be said not to be independent of total sports demand. As such, as noted by other authors, an optimal level of doping may exist [21]. Still, as our results indicate, standardizing doping tests as well as punishments (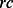 kept constant) makes sense as it reduces complexity in analysing doping games.

The popular version of Theorem 2 as depicted in equation (12) is interesting. This equation tells us that the region of unpredictability increases with the number of players in the game. To some extent this could indicate that keeping the number of players low actually is a good idea. Again, we should take care in pulling the policy implication string too long, but to fight doping, being able to predict athletes’ choices regarding doping seems evident. As such, a smaller number of competitors should (at least theoretically) be better than a larger number.

A practical example might be interesting to judge. Modern cross country skiing has undergone a major structural change the latter years. To a great extent, the classical competition structure of interval starts have been replaced by an increasing number of common (mass) start events. The actual reason for this changer is of course labelled on demand and TV-viewers - who presumably prefer to see a competition where the first man crossing the finish line is the winner. The fact that the time spent in mass start events is significantly shorter is of course also judged positive when competing to get TV-time. In an interval start event, even though you principally compete with the same number of competitors, your actual number of real competitors (based on your performance quality compared to the rest of the heat) is in practice way below the total number of participants. However, in a mass start event, the number of possible winners increase dramatically, meaning that you efficiently compete with a much larger group. Hence, based on this argument, if doping is to be controlled, this development is perhaps not the best. Still, again, this is a totality, and doping neither could nor should be fought regardless of possible adverse demand effects. This underlines the complexity of the task at hand.

Finally, the role of the prize function becomes evident when a doping game is extended from a two-player to an 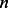-player game. Our results in Table 1 show clearly that the shape of this function is an important determinant for existence of various Nash equilibria. To some extent, this is obvious, as a two-player game can only contain a linear prize function. The possibility of dividing prizes egalitarian or not opens up when the participation number is larger than 2. Still, we feel that the results we have derived (even if they are not perfectly complete) warrant greater caution from sport officials when it comes to how prize functions are designed. To some extent, other research points at a similar problem - see for instance [26] and [27].

### Suggestions for Further Research

Handling multi-player games is in general far more difficult, both model- and solution-wise compared to 2-player games. Definitely, this is the situation we have faced conducting this research. As a consequence, we have carefully chosen to make assumptions which may seem crude compared to reality. Assumptions 2), 6), 7) and 8) (see the section on Assumptions and basic modeling), as well as the inherent not explicitly mentioned assumptions of information completeness and non-existent prize re-awards [5], are all examples of conditions that may seem far from reality.

Assumption 2) - homogeneity of player’s ability is a prime candidate. Most two-player research - see e.g. [7], [12], [21] tend to make such an assumption initially and then at later stages relax it. From a practical point of view such an assumption may at least to a certain extent seem sensible in a 2-player setting, but far more dramatic in a multi-player setting. However, relaxing such an assumption - in a multi-player setting - provides significant added challenges in the modeling as well as in the analysis stage. The reason is relatively straightforward to explain. In a multi player heterogeneous player situation, players may naturally be separated through a common knowledge probability distribution. Such a probability distribution must be 2-dimensional in this setting, both in prize as well as player dimensions. As a consequence, any given player must have a probability distribution for all possible prizes and all prizes must be won by at least on player. This fact introduces two probabilistic (norming) constraints which turns out to create significant modelling problems. In addition, the effect of doping must be included as well, leading to a situation where the model itself must account for all possible drug allocations and probabilistic consequences for all possible combinations of players, prizes and drug taking habits.

Unfortunately, this is not the only problem involved in relaxing the homogeneity of player’s ability assumption. Note that the share existence of a common knowledge ability 2-dimensional probability distribution actually means that all players must know (and agree) on such a distribution and not only that, they must also know and agree on all possible (individual) drug effects. So, to sum up: relaxing this assumption leads, in our opinion, to an obvious question related to the inherent complete information assumption. In practice one would expect that an individual player would know more on his abilities as well as the effect of taking drugs than his opponents. As a consequence, a game of incomplete (and asymmetric) information becomes even more appropriate. This is, as we see it, obviously a candidate for further research.

Similar types of (of course not identical) problems emerge if other assumptions are to be relaxed. For instance a relaxation of 7) - introducing more than one drug and/or individual drug effects - creates totally different modelling environments. This also holds for an introduction of prize re-awards. Prize re-awards [5] relates to the practical fact that if a player is caught in doping activity certain costs emerge. Such costs are (at least in principle) accounted for in our model through the 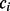 parameters. However, a doping verdict also has other consequences, namely that of prize re-awards - all other (not yet caught in doping) moves up one place on the prize-list. Surely, such mechanisms holds potential for significant game theoretic consequences, as reported in 2-player research [5], [7], [9], but we again feel that this subject needs projects of its own.

We have focused on modelling and solving a multi-player doping game. We have found interesting results related to the shape of prize functions and some results related to NE behaviour as a function of 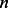. Obviously, we have not solved all possible modelling issues and it is relatively easy to criticise our models as being crude and unrealistic. Whilst it is true, we would welcome further investigations relaxing our assumptions and gaining even more insight into the mystery of doping.

## Supporting Information

Figure S1 The original pay-off matrix from [21].
(TIF)

Figure S2Nash equilibria in the three-player doping game as a function of the value of 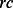. Each Nash equilibrium is represented by a box. Nash equilibria with the same 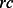 product are in the same column. 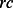 increases from left to right. An arrow points from one Nash equilibrium into another if changing 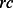 transforms a Nash equilibrium into another one.(TIF)

Figure S3Nash equilibria in the four-player doping game as a function of the value of 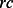. Each Nash equilibrium is represented by a box. Nash equilibria with the same 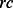 product are in the same column. 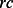 increases from left to right. An arrow points from one Nash equilibrium into another if changing 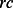 transforms a Nash equilibrium into another one.(TIF)

File S1 (PDF)
