# Supplementary material for: The Multi-Player Performance-Enhancing Drug Game
Source: PLoS One. 2013 May 17;8(5):e63306. doi: 10.1371/journal.pone.0063306 (PMC3656928; doi:10.1371/journal.pone.0063306)
Supplement: File S1 — (PDF) [file pone.0063306.s004.pdf]

Supporting Information: Analytical derivations for the multi-player doping game

Kjetil K. Haugen, Tamás Nepusz and Andrea Petróczi

April 10, 2013

Contents

1 The two-player performance-enhancing drug game as a special case 2

2 Proofs of Theorems in the main manuscript 3

3 Nash-equilibria in the three-player case 5

3.1 Pure strategies . . . 6

3.1.1 Case 1: everyone cheats (DDD) . . . 6

3.1.2 Case 2: two players cheat, one player plays fair (DDF) . . . 6

3.1.3 Case 3: one player cheats, two play fair (DFF) . . . 7

3.1.4 Case 4: everyone plays fair (FFF) . . . 7

3.2 Mixed strategies . . . 8

3.2.1 Case 5: everyone plays a mixed strategy (mmm) . . . 8

3.2.2 Case 6: one player cheats with certainty, others play a mixed strategy (Dmm) . . . 8

3.2.3 Case 7: one player plays fair with certainty, others play a mixed strategy (Fmm) . . . 9

3.2.4 Case 8: two players cheat with certainty, one plays a mixed strategy (DDm) 10

3.2.5 Case 9: two players play fair with certainty, one plays a mixed strategy (FFm) . . . 10

3.2.6 Case 10: one player plays fair, one player cheats, the third is mixed (DFm) 10

3.3 Summary of the three-player case . . . 11

4 Nash-equilibria in the four-player case 12

4.1 Pure strategies . . . 13

4.1.1 Case 1: everyone cheats (DDDD) . . . 13

4.1.2 Case 2: three players cheat, one player plays fair (DDDF) . . . 13

4.1.3 Case 3: two players cheat, two play fair (DDFF) . . . 14

4.1.4 Case 4: one player cheats, three play fair (DFFF) . . . 14

4.1.5 Case 5: everyone plays fair (FFFF) . . . 15

4.2 Mixed strategies . . . 15

4.2.1 Case 6: everyone plays a mixed strategy (mmmm) . . . 15

4.2.2 Case 7: three players cheat with certainty, one plays a mixed strategy (DDDm) . . . 16

4.2.3 Case 8: two players cheat with certainty, one plays fair with certainty, one plays a mixed strategy (DDFm) . . . 17

4.2.4 Case 9: one player cheats with certainty, two play fair with certainty, one plays a mixed strategy (DFFm) . . . 17
4.2.5 Case 10: three players play fair with certainty, one plays a mixed strategy (FFFm) . . . 18
4.2.6 Case 11: two players cheat with certainty, two play a mixed strategy (DDmm) . . . 19
4.2.7 Case 12: one player cheats with certainty, one plays fair with certainty, two play a mixed strategy (DFmm) . . . 20
4.2.8 Case 13: two players play fair with certainty, two play a mixed strategy (FFmm) . . . 22
4.2.9 Case 14: one player cheats with certainty, others play a mixed strategy (Dmmm) . . . 23
4.2.10 Case 15: one player plays fair with certainty, others play a mixed strategy (Fmmm) . . . 23
4.3 Summary of the four-player case . . . 24

5 Figures **27**

1 The two-player performance-enhancing drug game as a special case

In this section, we show how the multi-player doping game can be parametrised to reproduce the original two-player doping game of [1]; that is, we will check if the expanded model described in the Model set-up section of the main manuscript is able to regenerate the original performance-enhancing drug game in [1].

In order to mimic this model, we will need to assign correct values to the new set of parameters. It can be shown easily that the following parametrisation leads to this objective:

$$n = 2,\ a_1 = a,\ a_2 = 0,\ r_1 = r_2 = r \text{ and } c_1 = c_2 = c \tag{S1}$$

Given the parametric assumptions of Equation (S1), the variable d may take three values; 0, 1, or 2. If $d = 0$, nobody takes drugs and the expected payoff of players can be computed as:

$$\Pi_i^{\mathcal{ND}} = \frac{1}{2-0} \sum_{j=0+1}^{2} a_j = \frac{1}{2}(a+0) = \frac{1}{2}a \tag{S2}$$

In the case of two drug takers ($d = 2$), the expected payoff of players takes the form:

$$\Pi_i^{\mathcal{D}} = \frac{1}{2} \sum_{j=1}^{2} a_j - r_i c_i = \frac{1}{2}a - rc \tag{S3}$$

If $d = 1$, one player is "clean" while the other takes drugs. In this situation the payoff of the cheater becomes:

$$\Pi_i^{\mathcal{D}} = \frac{1}{1} \sum_{j=1}^{1} a_j - r_i c_i = a - rc \tag{S4}$$

while the payoff of the player that stays clean is:

$$\Pi_i^{\mathcal{ND}} = \frac{1}{2-1} \sum_{j=1+1}^{2} a_j = 1 \cdot 0 = 0 \tag{S5}$$

Now, comparing the results of (S2), (S3), (S4) and (S5) with the original pay-off structure of [1] a perfect correspondence is readily observed. (See Figure S1)

Consequently, our extended multi player model is shown to represent the two-player case correctly.

2 Proofs of Theorems in the main manuscript

Let us start from the expected payoff of player i when he cheats with probability p_i. Let $D^{(i)}$ denote the number of players taking doping, not counting player i itself (note that this is a random variable). The payoff function is then:

$$\Pi_i = p_i \left(\sum_{d=0}^{n-1} \mathbf{P}(D^{(i)} = d) \frac{\sum_{i=1}^{d+1} a_i}{d+1} - r_i c_i \right) + (1 - p_i) \left(\sum_{d=0}^{n-1} \mathbf{P}(D^{(i)} = d) \frac{\sum_{i=d+1}^{n} a_i}{n-d} \right)$$

where $A_d = \frac{\sum_{i=1}^{d} a_i}{d}$ is the average prize received by a player that cheated if there are d cheaters in total, and $\bar{A}_d = \frac{\sum_{i=d+1}^{n} a_i}{n-d}$ is the average prize received by players that played fair. By applying these definitions, the expression can be simplified as:

$$\begin{aligned} \Pi_i &= p_i \left(\sum_{d=0}^{n-1} \mathbf{P}(D^{(i)} = d) A_{d+1} - r_i c_i \right) + (1 - p_i) \left(\sum_{d=0}^{n-1} \mathbf{P}(D^{(i)} = d) \bar{A}_d \right) \\ &= p_i \sum_{d=0}^{n-1} \mathbf{P}(D^{(i)} = d) \left(A_{d+1} - \bar{A}_d \right) - p_i r_i c_i + \text{const.} \end{aligned}$$

Taking the derivative:

$$\frac{\partial \Pi_i}{\partial p_i} = \sum_{d=0}^{n-1} \mathbf{P}(D^{(i)} = d) \left(A_{d+1} - \bar{A}_d \right) - r_i c_i \tag{S6}$$

Since p_i is constrained to the range [0; 1], the condition for a Nash equilibrium is that for each player, exactly one of the following three cases must hold:

1. $\frac{\partial \Pi_i}{\partial p_i} = 0$ and $0 \leq p_i \leq 1$
2. $\frac{\partial \Pi_i}{\partial p_i} > 0$ and $p_i = 1$
3. $\frac{\partial \Pi_i}{\partial p_i} < 0$ and $p_i = 0$

Note that if we expand the sum in the derivative and spell out the $\mathbf{P}(D^{(i)} = d)$ probabilities exactly, we would find that the derivative of Π_i w.r.t. p_i depends only on p_j where $j \neq i$, and their form is equivalent apart from the indices of the p_i's involved. Therefore, when we evaluate combinations of the three conditions outlined above, we only have to decide *how many* players will be treated according to the first, second or third conditions, without loss of generality.

Theorem 1. *Given an instance of the n-player doping game, a common rc product for all players, and a linear prize structure (i.e. a common $b = a_1 - a_2$), the Nash-equilibria are as follows:*

1. *Everyone cheats when $rc < \frac{n-1}{2} b$.*

2. *Everyone plays fair when* $rc > \frac{n-1}{2}b$.

3. *Any pure or mixed strategy when* $rc = \frac{n-1}{2}b$.

Proof. When the prize structure is linear, we know that $a_1 - a_2 = a_3 - a_2 = \ldots$, and that $a_i = a_1 - (i-1)b$ where b is defined as $a_1 - a_2$. This implies that

$$\begin{aligned} A_k &= \frac{\sum_{i=1}^{k} a_i}{k} = a_1 - \frac{b}{k}\sum_{i=1}^{k-1} i = a_1 - \frac{b(k-1)}{2} \\ \bar{A}_k &= \frac{\sum_{i=k+1}^{n} a_i}{n-k} = a_1 - \frac{b}{n-k}\sum_{i=k}^{n-1} i = a_1 - \frac{b(n-k+1)}{2} \\ A_{k+1} - \bar{A}_k &= b\frac{n-1}{2} \end{aligned}$$

The general derivative in Equation (S6) is then simplified to:

$$\frac{\partial \Pi_i}{\partial p_i} = \sum_{d=0}^{n-1} \mathbf{P}(D^{(i)} = d) b\frac{n-1}{2} - rc = b\frac{n-1}{2} - rc$$

It is now clear that the derivatives are independent of i, i.e. they are the same for all the players. The derivatives are zero when $rc = b\frac{n-1}{2}$, and since this constraint imposes no restriction on the probabilities, it follows that any pure or mixed strategy is a Nash equilibrium when $rc = b\frac{n-1}{2}$ and the prize structure is linear. $rc < b\frac{n-1}{2}$ implies a positive derivative for every player, leading to a situation where everyone cheats. Similarly, $rc > b\frac{n-1}{2}$ yields a negative derivative and everyone will play fair. □

Theorem 2. *Given an instance of the n-player doping game and a common rc product for all players, the sufficient and necessary condition for the "k players cheat and n − k players play fair" pure strategy to be a Nash-equilibrium is as follows:*

$$A_{k+1} - \bar{A}_k \le rc \le A_k - \bar{A}_{k-1}$$

where A_{n+1} *and* $\bar{A}_{-1}$ *are defined to be* $-\infty$.

Proof. Without loss of generality, let us assume that players 1, 2, ..., k cheat and the rest play fair. When we are in a Nash equilibrium and k players cheat with probability 1, this means that k players have no incentive for switching their strategy because their payoff function is locally maximal. Similarly, for the remaining $n-k$ players, they also have no incentive to switch because their payoff function is locally maximal. If there were no limits on the range of p_i (i.e. the probability of cheating), the equations were as follows:

$$\frac{\partial \Pi_i}{\partial p_i} = 0 \qquad \text{for every } i$$

This means that the derivatives of the payoff function are zeros for everyone. However, since p_i is bounded from above by 1 and from below by zero, the derivative of the payoff function of cheaters may actually be larger than or equal to zero, where being larger means that the player would try to cheat even more frequently if that were possible. A similar train of thought shows that the derivative of the payoff function of non-cheaters may actually be smaller than or equal to zero, meaning that they would try to play even more fairly if that were possible. This yields the following conditions to start out from:

$$\frac{\partial \Pi_i}{\partial p_i} \geq 0 \qquad \text{if } i \leq k$$
$$\frac{\partial \Pi_i}{\partial p_i} \leq 0 \qquad \text{if } i > k$$

We use Equation (S6) to simplify them as follows:

1. For player i where $i \leq k$, the player is cheating, and the number of other players who are cheating is $k-1$ with certainty (since all p_i probabilities are either zeros or ones). Substituting $d = k-1$ into Equation (S6) gives us $\left(A_k - \bar{A}_{k-1}\right) - r_i c_i \geq 0$.

2. For player i where $i > k$, the player is playing fair, and the number of other players who are cheating is k with certainty (since all p_i probabilities are either zeros or ones). Substituting $d = k$ into Equation (S6) gives us $\left(A_{k+1} - \bar{A}_k\right) - r_i c_i \leq 0$.

We then assume a common rc product and move $r_i c_i$ to the right hand side to obtain the final equation:

$$A_{k+1} - \bar{A}_k \leq rc \leq A_k - \bar{A}_{k-1}$$

This concludes our proof. □

3 Nash-equilibria in the three-player case

To describe the various Nash-equilibria we will find, we will use a simple encoding scheme where each letter describes the behaviour of one player. Each letter may be D (doping with certainty), F (plays fair with certainty), m (mixed strategy) or $*$ (any pure or mixed strategy). For instance, DDF describes a strategy where two players cheat and the third plays fair all the time, while mmD is a strategy where two players play mixed strategies and the third cheats all the time. The order of letters does not matter, mmD is the same as mDm or Dmm. The difference between m and $*$ is subtle but significant: when a player is marked by m, it means that the player will assume a mixed strategy with an exact probability for cheating, while $*$ means that the player may take *any* pure or mixed strategy with any probabiity for cheating without affecting its own payoff function.

In the three-player case, we know the following:

$$A_0 = 0 \qquad A_1 = a_1 \qquad A_2 = \frac{a_1 + a_2}{2} \qquad A_3 = \frac{a_1 + a_2 + a_3}{3}$$
$$\bar{A}_0 = \frac{a_1 + a_2 + a_3}{3} \qquad \bar{A}_1 = \frac{a_2 + a_3}{2} \qquad \bar{A}_2 = a_3 \qquad \bar{A}_3 = 0$$
$$A_1 - \bar{A}_0 = \frac{2a_1 - a_2 - a_3}{3} \qquad A_2 - \bar{A}_1 = \frac{a_1 - a_3}{2} \qquad A_3 - \bar{A}_2 = \frac{a_1 + a_2 - 2a_3}{3}$$

We also know that

$$\mathbf{P}(D^{(1)} = 0) = (1-p_2)(1-p_3) = 1 - p_2 - p_3 + p_2 p_3$$
$$\mathbf{P}(D^{(1)} = 1) = p_2(1-p_3) + p_3(1-p_2) = p_2 + p_3 - 2p_2 p_3$$
$$\mathbf{P}(D^{(1)} = 2) = p_2 p_3$$

The derivative is then

$$
\begin{aligned}
\frac{\partial \Pi_1}{\partial p_1} &= (1-p_2-p_3+p_2p_3)\frac{2a_1-a_2-a_3}{3}+(p_2+p_3-2p_2p_3)\frac{a_1-a_3}{2}+p_2p_3\frac{a_1+a_2-2a_3}{3}-r_1c_1 \\
&= \frac{2a_1-a_2-a_3}{3}+(p_2+p_3)\frac{2a_2-a_1-a_3}{6}+p_2p_3\times 0-r_1c_1 \\
&= (p_2+p_3)\frac{b_2-b_1}{6}+\frac{2b_1+b_2}{3}-r_1c_1
\end{aligned}
$$

where $b_i = a_i - a_{i+1}$. Note that the b_i's are just an alternate representation of the prize structure as b_i is the difference between the ith and the $i+1$th prize. The remaining derivatives are the same but with different indices for p_i, r_i and c_i, but the indices for b_i stay the same:

$$
\begin{aligned}
\frac{\partial \Pi_2}{\partial p_2} &= (p_1+p_3)\frac{b_2-b_1}{6}+\frac{2b_1+b_2}{3}-r_1c_1 \\
\frac{\partial \Pi_3}{\partial p_3} &= (p_1+p_2)\frac{b_2-b_1}{6}+\frac{2b_1+b_2}{3}-r_1c_1
\end{aligned}
$$

3.1 Pure strategies

3.1.1 Case 1: everyone cheats (DDD)

This means that all the three players are according to condition 2. We have to decide whether all the derivatives are non-negative (given that all cheat, $p_1 = p_2 = p_3 = 1$):

$$
\begin{aligned}
2b_2+b_1-3r_1c_1 &\geq 0 \\
2b_2+b_1-3r_2c_2 &\geq 0 \\
2b_2+b_1-3r_3c_3 &\geq 0
\end{aligned}
$$

Therefore, this particular configuration (i.e. everyone cheats) is a Nash equilibrium if and only if $\frac{2b_2+b_1}{3} \geq \max_i r_ic_i$. Increasing r_ic_i above $\frac{2b_2+b_1}{3}$ for at least one player eradicates this configuration.

A special case is the case of linear prize structures where $b_1 = b_2 = \cdots = b_{n-1} = b$, leading to $b \geq \max r_ic_i$. In other words, if the expected loss of player i when cheating is less than the difference between two consecutive places in the prize ladder, then the player will cheat.

3.1.2 Case 2: two players cheat, one player plays fair (DDF)

Without loss of generality, we can assume that players 1 and 2 cheat and player 3 plays fair. Thus, $p_1 + p_2 = 2$, $p_1 + p_3 = 1$ and $p_2 + p_3 = 1$. The equations to check are:

$$
\begin{aligned}
\frac{b_1+b_2}{2}-r_1c_1 &\geq 0 \\
\frac{b_1+b_2}{2}-r_2c_2 &\geq 0 \\
\frac{2b_2+b_1}{3}-r_3c_3 &\leq 0
\end{aligned}
$$

Here it becomes easier to assume that r_ic_i is independent of i, yielding:

$$\frac{b_1 + b_2}{2} \geq rc \geq \frac{2b_2 + b_1}{3}$$

The above equation may hold only when $b_1 \geq b_2$; in other words, when the prize structure is convex or linear. If we assume the case of linear prize structure (i.e. $b_1 = b_2 = b$), we obtain $b \geq rc \geq b$, which can happen only if $rc = b$. We will see later that $rc = b$ collapses the game into a configuration where all the strategies are equivalent anyway.

3.1.3 Case 3: one player cheats, two play fair (DFF)

Again, without loss of generality, we can assume that player 1 cheats and players 2 and 3 play fair. Thus, $p_1 + p_2 = 1$, $p_1 + p_3 = 1$ and $p_2 + p_3 = 0$. The equations are:

$$\begin{aligned} \frac{2b_1 + b_2}{3} - r_1 c_1 &\geq 0 \\ \frac{b_1 + b_2}{2} - r_2 c_2 &\leq 0 \\ \frac{b_1 + b_2}{2} - r_3 c_3 &\leq 0 \end{aligned}$$

Assuming $r_i c_i = rc$:

$$\frac{2b_1 + b_2}{3} \geq rc \geq \frac{b_1 + b_2}{2}$$

Note that this can hold only when $b_1 \leq b_2$; in other words, when the prize structure is concave or linear.

3.1.4 Case 4: everyone plays fair (FFF)

In this case, we have $p_1 = p_2 = p_3 = 0$, which leads to

$$\begin{aligned} \frac{2b_1 + b_2}{3} - r_1 c_1 &\leq 0 \\ \frac{2b_1 + b_2}{3} - r_2 c_2 &\leq 0 \\ \frac{2b_1 + b_2}{3} - r_3 c_3 &\leq 0 \end{aligned}$$

Therefore, this particular configuration (i.e. everyone plays fair) is a Nash equilibrium if and only if $\frac{2b_1+b_2}{3} \leq \min r_i c_i$. Decreasing $r_i c_i$ below $\frac{2b_1+b_2}{3}$ for at least one player eradicates this configuration.

A special case is the case of linear prize structures which leads to $b \leq \min r_i c_i$. In other words, if the expected loss of player i when cheating is greater than the difference between two consecutive places in the prize ladder, then the player will play fair.

Let us take a step back now and examine what we have observed so far. The presence or absence of various pure Nash-equilibria depend on where the value of the rc product falls compared to $\frac{2b_1+b_2}{3}$, $\frac{b_1+b_2}{2}$ and $\frac{b_1+2b_2}{3}$. Furthermore, note that the thresholds are also equal to $\bar{A}_1 - \bar{A}_0$, $\bar{A}_2 - \bar{A}_1$ and $\bar{A}_3 - \bar{A}_2$. We will see that these thresholds will also determine the behaviour of the model when we allow for mixed strategies as well.

3.2 Mixed strategies

3.2.1 Case 5: everyone plays a mixed strategy (mmm)

In this configuration, all the partial derivatives must be equal to zero:

$$\begin{aligned}
(p_2+p_3)\frac{b_2-b_1}{6}+\frac{2b_1+b_2}{3} &= r_1c_1 \\
(p_1+p_3)\frac{b_2-b_1}{6}+\frac{2b_1+b_2}{3} &= r_2c_2 \\
(p_1+p_2)\frac{b_2-b_1}{6}+\frac{2b_1+b_2}{3} &= r_3c_3
\end{aligned}$$

When $b_1 = b_2$ (i.e. the prize structure is linear), the first term is zero and we recover cases 1 and 4, respectively. Let us therefore assume that $b_1 \neq b_2$. This leads to:

$$\begin{aligned}
p_2+p_3 &= \frac{6r_1c_1-4b_1-2b_2}{b_2-b_1} \\
p_1+p_3 &= \frac{6r_2c_2-4b_1-2b_2}{b_2-b_1} \\
p_1+p_2 &= \frac{6r_3c_3-4b_1-2b_2}{b_2-b_1}
\end{aligned}$$

Again, assuming that $r_ic_i = rc$, we obtain

$$p_1 = p_2 = p_3 = 3\left(\frac{rc-b_1}{b_2-b_1}\right)-1$$

We also have to ensure that $0 \leq p_i \leq 1$, otherwise this solution would not exist. Therefore, this Nash equilibrium occurs if and only if

$$\frac{1}{3} \leq \frac{rc-b_1}{b_2-b_1} \leq \frac{2}{3}$$

which is equivalent to

$$\frac{2b_1+b_2}{3} \leq rc \leq \frac{b_1+2b_2}{3}$$

where we notice the same thresholds again as the ones we have seen for the pure case.

3.2.2 Case 6: one player cheats with certainty, others play a mixed strategy (Dmm)

Without loss of generality, assume that player 1 cheats with certainty. The equations are:

$$\begin{aligned}
(p_2+p_3)\frac{b_2-b_1}{6}+\frac{2b_1+b_2}{3} &\geq r_1c_1 \\
(1+p_3)\frac{b_2-b_1}{6}+\frac{2b_1+b_2}{3} &= r_2c_2 \\
(1+p_2)\frac{b_2-b_1}{6}+\frac{2b_1+b_2}{3} &= r_3c_3
\end{aligned}$$

Again, when $b_1 = b_2$, the conditions recover case 3, so let us assume that $b_1 \neq b_2$. We then have to distinguish two cases.

The first case is when $b_1 > b_2$, i.e. a convex prize structure. In this case, $b_2 - b_1 < 0$, so the relation will turn in the first equation as we divide by a negative number:

$$\begin{aligned} p_2 + p_3 &\leq \frac{6r_1c_1 - 4b_1 - 2b_2}{b_2 - b_1} \\ p_3 &= \frac{6r_2c_2 - 3b_1 - 3b_2}{b_2 - b_1} \\ p_2 &= \frac{6r_3c_3 - 3b_1 - 3b_2}{b_2 - b_1} \end{aligned}$$

Assuming a common rc, this means that $p_2 = p_3 = \frac{6rc}{b_2-b_1} - 3\frac{b_1+b_2}{b_2-b_1}$, but since we also have to satisfy the first equation, this will happen only if

$$\begin{aligned} \frac{12rc - 6b_1 - 6b_2}{b_2 - b_1} &\leq \frac{6rc - 4b_1 - 2b_2}{b_2 - b_1} \\ 12rc - 6b_1 - 6b_2 &\geq 6rc - 4b_1 - 2b_2 \\ rc &\geq \frac{b_1 + 2b_2}{3} \end{aligned}$$

We also have to ensure that p_2 and p_3 are between 0 and 1:

$$\begin{aligned} 0 \leq &\quad \tfrac{6rc-3b_1-3b_2}{b_2-b_1} &\leq 1 \\ 0 \geq &\quad 6rc - 3b_1 - 3b_2 &\geq b_2 - b_1 \\ \frac{b_1 + b_2}{2} \geq &\quad rc &\geq \frac{b_1 + 2b_2}{3} \end{aligned}$$

The other case is when $b_2 > b_1$, i.e. the prize structure is concave. In this case, the relation mark does not turn, but since p_2 and p_3 were determined from the second and third equations that do not contain the relation mark, the solution will be the same. Substituting p_2 and p_3 back into the first equation yields $rc \geq \frac{b_1+2b_2}{3}$. Therefore, this Nash equilibrium will appear if and only if

$$\frac{b_1 + 2b_2}{3} \leq rc \leq \frac{b_1 + b_2}{2}$$

3.2.3 Case 7: one player plays fair with certainty, others play a mixed strategy (Fmm)

Without loss of generality, assume that player 1 plays fair with certainty. The equations are:

$$\begin{aligned} (p_2 + p_3)\frac{b_2 - b_1}{6} + \frac{2b_1 + b_2}{3} &\leq r_1c_1 \\ p_3\frac{b_2 - b_1}{6} + \frac{2b_1 + b_2}{3} &= r_2c_2 \\ p_2\frac{b_2 - b_1}{6} + \frac{2b_1 + b_2}{3} &= r_3c_3 \end{aligned}$$

With a common rc, the second and third equations yield $p_2 = p_3 = \frac{6rc-4b_1-2b_2}{b_2-b_1}$, and to satisfy the first one, we also need $rc \leq \frac{2b_1+b_2}{3}$. Since p_2 and p_3 must be between zero and one, the complete set of conditions is:

$$\frac{b_1+b_2}{2} \leq rc \leq \frac{2b_1+b_2}{3}$$

3.2.4 Case 8: two players cheat with certainty, one plays a mixed strategy (DDm)

Without loss of generality, assume that players 1 and 2 cheat with certainty. The equations are:

$$\begin{aligned}
(1+p_3)\frac{b_2-b_1}{6}+\frac{2b_1+b_2}{3} &\geq r_1c_1 \\
(1+p_3)\frac{b_2-b_1}{6}+\frac{2b_1+b_2}{3} &\geq r_2c_2 \\
\frac{2b_2+b_1}{3} &= r_3c_3
\end{aligned}$$

Assuming a common rc, the third equation clearly places a constraint on the occurrence of this NE – it will occur only for a particular value of rc. In that case, the first two equations will prescribe an upper or a lower bound on p_3, depending on the sign of $b_2 - b_1$.

When $b_2 > b_1$, there will be a lower bound on p_3, i.e. $p_3 > \frac{6rc}{b_2-b_1} - 3\frac{b_1+b_2}{b_2-b_1}$, but since we know rc exactly from the third equation, this yields $p_3 \geq 1$. Since p_3 is a probability, the only allowed value is $p_3 = 1$, which is in fact case 1 (three players cheat with certainty). Thus, case 8 does not exist for concave prize structures.

When $b_2 < b_1$, there will be a trivial upper bound on p_3, i.e. $p_3 \leq 1$, which is not a restriction on p_3, meaning that case 8 is better marked as the DD$*$ case.

3.2.5 Case 9: two players play fair with certainty, one plays a mixed strategy (FFm)

Without loss of generality, assume that players 1 and 2 play fair with certainty. The equations are:

$$\begin{aligned}
p_3\frac{b_2-b_1}{6}+\frac{2b_1+b_2}{3} &\leq r_1c_1 \\
p_3\frac{b_2-b_1}{6}+\frac{2b_1+b_2}{3} &\leq r_2c_2 \\
\frac{2b_1+b_2}{3} &= r_3c_3
\end{aligned}$$

Similarly to case 8, case 9 will either collapse to case 4 (FFF) when $b_2 > b_1$, or will collapse to FF$*$ if $b_2 < b_1$.

3.2.6 Case 10: one player plays fair, one player cheats, the third is mixed (DFm)

Without loss of generality, assume that player 1 plays fair, player 2 cheats and player 3 is mixed. The equations are:

$$
\begin{aligned}
(1+p_3)\frac{b_2-b_1}{6}+\frac{2b_1+b_2}{3} &\leq r_1c_1\\
p_3\frac{b_2-b_1}{6}+\frac{2b_1+b_2}{3} &\geq r_2c_2\\
\frac{b_1+b_2}{2} &= r_3c_3
\end{aligned}
$$

Let us assume a common rc:

$$
\begin{aligned}
p_3(b_2-b_1) &\leq 0\\
(p_3-1)(b_2-b_1) &\geq 0
\end{aligned}
$$

If $b_1 < b_2$, the only solution is $p_3 = 0$, collapsing this case into case 2 (DDF). If $b_1 > b_2$, any p_4 satisfies the equations, making it equivalent to DF$*$.

3.3 Summary of the three-player case

All the conditions we have obtained for rc depended on three thresholds: $\frac{2b_1+b_2}{3}$, $\frac{b_1+b_2}{2}$ and $\frac{b_1+2b_2}{3}$. These are also equal to $A_1-\bar{A}_0$, $A_2-\bar{A}_1$ and $A_3-\bar{A}_2$. The behaviour of the model will depend on the relation of rc to these thresholds. First, let us assume that $b_1 < b_2$, i.e. the prize structure is concave. In this case, we have the following:

rc	Cases of Nash-equilibria
Less than $A_3-\bar{A}_2$	DDD
Exactly $A_3-\bar{A}_2$	DDD, DDF
Between $A_3-\bar{A}_2$ and $A_2-\bar{A}_1$	DDF, Dmm, mmm
Exactly $A_2-\bar{A}_1$	DDF, DFF, mmm with $p=0.5$
Between $A_2-\bar{A}_1$ and $A_1-\bar{A}_0$	DFF, Fmm, mmm
Exactly $A_1-\bar{A}_0$	DFF, FFF
Greater than $A_1-\bar{A}_0$	FFF

When the prize structure is convex, b_1 is larger than b_2, leading to the following cases:

rc	Cases of Nash-equilibria
Less than $A_3 - \bar{A}_2$	DDD
Exactly $A_3 - \bar{A}_2$	DD$*$
Between $A_3 - \bar{A}_2$ and $A_2 - \bar{A}_1$	DDF, Dmm, mmm
Exactly $A_2 - \bar{A}_1$	DF$*$, mmm with $p = 0.5$
Between $A_2 - \bar{A}_1$ and $A_1 - \bar{A}_0$	DFF, Fmm, mmm
Exactly $A_1 - \bar{A}_0$	FF$*$
Greater than $A_1 - \bar{A}_0$	FFF

For linear prize structures, $b_1 = b_2 = b = A_2 - \bar{A}_1$ and we obtain:

rc	Cases of Nash-equilibria
Less than $A_2 - \bar{A}_1$	DDD
Exactly $A_2 - \bar{A}_1$	$* * *$
Greater than $A_2 - \bar{A}_1$	FFF

Finally, we present an alternative representation of the the different types of Nash equilibria on Figure S2. On this figure, each Nash equilibrium is represented by a box and Nash equilibria corresponding to the same rc value are arranged in the same column. The value of the rc product increases from left to right. Arrows denote the "transition" of one type of Nash equilibrium into another (for instance, a DDD type Nash equilibrium into Dmm) as the value of rc changes. The upper panel contains the case of concave prize functions while the lower panel contains the convex case.

4 Nash-equilibria in the four-player case

In the four-player case, we know the following:

$$A_0 = 0 \quad A_1 = a_1 \quad A_2 = \frac{a_1 + a_2}{2} \quad A_3 = \frac{a_1 + a_2 + a_3}{3} \quad A_4 = \frac{a_1 + a_2 + a_3 + a_4}{4}$$

$$\bar{A}_0 = \frac{a_1 + a_2 + a_3 + a_4}{4} \quad \bar{A}_1 = \frac{a_2 + a_3 + a_4}{3} \quad \bar{A}_2 = \frac{a_3 + a_4}{2} \quad \bar{A}_3 = a_4 \quad \bar{A}_4 = 0$$

$$A_1 - \bar{A}_0 = \frac{3a_1 - a_2 - a_3 - a_4}{4} = \frac{3b_1 + 2b_2 + b_3}{4}$$

$$A_2 - \bar{A}_1 = \frac{3a_1 + a_2 - 2a_3 - 2a_4}{6} = \frac{3b_1 + 4b_2 + 2b_3}{6}$$

$$A_3 - \bar{A}_2 = \frac{2a_1 + 2a_2 - a_3 - 3a_4}{6} = \frac{2b_1 + 4b_2 + 3b_3}{6}$$

$$A_4 - \bar{A}_3 = \frac{a_1 + a_2 + a_3 - 3a_4}{4} = \frac{b_1 + 2b_2 + 3b_3}{4}$$

We also know that

$$
\begin{aligned}
\mathbf{P}(D^{(1)}=0) &= (1-p_2)(1-p_3)(1-p_4) \\
&= 1-p_2-p_3-p_4+p_2p_3+p_2p_4+p_3p_4-p_2p_3p_4 \\
\mathbf{P}(D^{(1)}=1) &= p_2(1-p_3)(1-p_4)+(1-p_2)p_3(1-p_4)+ \\
&\quad (1-p_2)(1-p_3)p_4 \\
&= p_2-p_2p_3-p_2p_4+p_2p_3p_4+p_3-p_2p_3-p_3p_4+p_2p_3p_4+ \\
&\quad p_4-p_2p_4-p_3p_4+p_2p_3p_4 \\
&= p_2+p_3+p_4-2p_2p_3-2p_2p_4-2p_3p_4+3p_2p_3p_4 \\
\mathbf{P}(D^{(1)}=2) &= p_2p_3(1-p_4)+p_2(1-p_3)p_4+(1-p_2)p_3p_4 \\
&= p_2p_3+p_2p_4+p_3p_4-3p_2p_3p_4 \\
\mathbf{P}(D^{(1)}=3) &= p_2p_3p_4
\end{aligned}
$$

The derivative of the payoff function Π_1 with respect to p_1 is then

$$
\begin{aligned}
\frac{\partial \Pi_1}{\partial p_1} &= \frac{b_3-2b_2+b_1}{12}(p_2p_3+p_2p_4+p_3p_4)+ \\
&\quad \frac{b_3+2b_2-3b_1}{12}(p_2+p_3+p_4)+ \\
&\quad \frac{3b_1+2b_2+b_3}{4}-r_1c_1
\end{aligned}
$$

4.1 Pure strategies

4.1.1 Case 1: everyone cheats (DDDD)

We have to decide whether all the derivatives are non-negative (given that all cheat, $p_1=p_2=p_3=p_4=1$):

$$
\begin{aligned}
b_1+2b_2+3b_3-4r_1c_1 &\geq 0 \\
b_1+2b_2+3b_3-4r_2c_2 &\geq 0 \\
b_1+2b_2+3b_3-4r_3c_3 &\geq 0 \\
b_1+2b_2+3b_3-4r_4c_4 &\geq 0
\end{aligned}
$$

Therefore, this particular configuration is a Nash equilibrium if and only if $\frac{b_1+2b_2+3b_3}{4} \geq \max_i r_ic_i$; note that the left hand side is equal to $A_4-\bar{A}_3$, which echoes the rule we have seen for the three player case, where the threshold was $A_3-\bar{A}_2$.

4.1.2 Case 2: three players cheat, one player plays fair (DDDF)

Without loss of generality, we can assume that players 1, 2 and 3 cheat and player 4 plays fair. Thus, $p_1p_2=p_2p_3=p_1p_3=1$, $p_1p_4=p_2p_4=p_3p_4=0$, $p_1+p_2+p_3=3$, $p_1+p_2+p_4=p_1+p_3+p_4=p_2+p_3+p_4=2$. The equations to check are:

$$\begin{aligned}
\frac{2b_1+4b_2+3b_3}{6} - r_1c_1 &\geq 0 \\
\frac{2b_1+4b_2+3b_3}{6} - r_2c_2 &\geq 0 \\
\frac{2b_1+4b_2+3b_3}{6} - r_3c_3 &\geq 0 \\
\frac{b_1+2b_2+3b_3}{4} - r_4c_4 &\leq 0
\end{aligned}$$

Assuming a common rc yields:

$$\frac{2b_1+4b_2+3b_3}{6} > rc > \frac{b_1+2b_2+3b_3}{4}$$

where the left hand side is equal to $A_3 - \bar{A}_2$ and the right hand side is equal to $A_4 - \bar{A}_3$. Note that for linear prize structures, this can happen only if $rc = \frac{3}{2}b$.

4.1.3 Case 3: two players cheat, two play fair (DDFF)

Without loss of generality, we can assume that players 1, 2 cheat and players 3 and 4 play fair. The equations to check are:

$$\begin{aligned}
\frac{3b_1+4b_2+2b_3}{6} - r_1c_1 &\geq 0 \\
\frac{3b_1+4b_2+2b_3}{6} - r_2c_2 &\geq 0 \\
\frac{2b_1+4b_2+3b_3}{6} - r_3c_3 &\geq 0 \\
\frac{2b_1+4b_2+3b_3}{6} - r_4c_4 &\leq 0
\end{aligned}$$

Assuming a common rc yields:

$$\frac{3b_1+4b_2+2b_3}{6} \geq rc \geq \frac{2b_1+4b_2+3b_3}{6}$$

where the left hand side is equal to $A_2 - \bar{A}_1$ and the right hand side is equal to $A_3 - \bar{A}_2$. For linear prize structures, this will collapse into $rc = \frac{3}{2}b$ as above.

4.1.4 Case 4: one player cheats, three play fair (DFFF)

Without loss of generality, we can assume that player 1 cheats and players 2, 3 and 4 play fair. The equations to check are:

$$\begin{aligned}
\frac{3b_1+2b_2+b_3}{4} - r_1c_1 &\geq 0 \\
\frac{3b_1+4b_2+2b_3}{6} - r_2c_2 &\leq 0 \\
\frac{3b_1+4b_2+2b_3}{6} - r_3c_3 &\leq 0 \\
\frac{3b_1+4b_2+2b_3}{6} - r_4c_4 &\leq 0
\end{aligned}$$

Assuming a common rc yields:

$$\frac{3b_1 + 2b_2 + b_3}{4} \geq rc \geq \frac{3b_1 + 4b_2 + 2b_3}{6}$$

where the left hand side is equal to $A_1 - \bar{A}_0$ and the right hand side is equal to $A_2 - \bar{A}_1$. For linear prize structures, this will collapse into $rc = \frac{3}{2}b$ as above.

4.1.5 Case 5: everyone plays fair (FFFF)

We have to decide whether all the derivatives are negative (given that all play fair, $p_1 = p_2 = p_3 = p_4 = 0$):

$$\begin{aligned}
3b_1 + 2b_2 + b_3 - 4r_1c_1 &\leq 0 \\
3b_1 + 2b_2 + b_3 - 4r_2c_2 &\leq 0 \\
3b_1 + 2b_2 + b_3 - 4r_3c_3 &\leq 0 \\
3b_1 + 2b_2 + b_3 - 4r_4c_4 &\leq 0
\end{aligned}$$

Therefore, this particular configuration is a Nash equilibrium if and only if $\frac{3b_1+2b_2+b_3}{4} \leq \min_i r_ic_i$; note that the left hand side is equal to $A_1 - \bar{A}_0$, which echoes the rule we have seen in case 4 of the three-player case.

Let us take a step back now again and examine what we have observed so far. The presence or absence of various pure Nash-equilibria depend on where the value of the rc product falls compared to $\frac{3b_1+2b_2+b_3}{4}$, $\frac{3b_1+4b_2+2b_3}{6}$, $\frac{2b_1+4b_2+3b_3}{6}$ and $\frac{b_1+2b_2+3b_3}{4}$. Furthermore, note that the thresholds are also equal to $A_1 - \bar{A}_0$, $A_2 - \bar{A}_1$, $A_3 - \bar{A}_2$ and $A_4 - \bar{A}_3$. We will encounter these thresholds later when we allow for mixed strategies as well.

4.2 Mixed strategies

4.2.1 Case 6: everyone plays a mixed strategy (mmmm)

In this configuration, all the partial derivatives must be equal to zero. Let's see where this leads:

$$\begin{aligned}
(p_2p_3 + p_2p_4 + p_3p_4)\frac{b_3 - 2b_2 + b_1}{12} &+ \\
(p_2 + p_3 + p_4)\frac{b_3 + 2b_2 - 3b_1}{12} &+ \\
\frac{3b_1 + 2b_2 + b_3}{4} &= r_1c_1
\end{aligned}$$

and so on for r_2c_2, r_3c_3 and r_4c_4. Here we assume a common rc again. For sake of simplicity, let us denote $b_3 - 2b_2 + b_1$ with C_1, $b_3 + 2b_2 - 3b_1$ with C_2 and $3b_1 + 2b_2 + b_3$ with C_3:

$$\begin{aligned}
(p_2p_3 + p_2p_4 + p_3p_4)C_1 + (p_2 + p_3 + p_4)C_2 &= 12rc - 3C_3 \\
(p_1p_3 + p_1p_4 + p_3p_4)C_1 + (p_1 + p_3 + p_4)C_2 &= 12rc - 3C_3 \\
(p_1p_2 + p_1p_4 + p_2p_4)C_1 + (p_1 + p_2 + p_4)C_2 &= 12rc - 3C_3 \\
(p_1p_2 + p_1p_3 + p_2p_3)C_1 + (p_1 + p_2 + p_3)C_2 &= 12rc - 3C_3
\end{aligned}$$

Because of the common rc, all the players are equivalent and all the equations are symmetric so we have no reason to assume that $p_1 \neq p_2$ or $p_2 \neq p_3$ or $p_3 \neq p_4$. (Proof by contradiction:

suppose that $p_1 \neq p_2$; in this case, the left hand side of the first two equations are different but the right hand side are the same, so they cannot be both satisfied). Switching to a common p gives us a single quadratic equation:

$$C_1 p^2 + C_2 p + C_3 - 4rc = 0$$

and the two solutions are:

$$p = \frac{-C_2 \pm \sqrt{C_2^2 - 4C_1C_3 + 16C_1rc}}{2C_1} = -\frac{C_2}{2C_1} \pm \sqrt{\left(\frac{C_2}{2C_1}\right)^2 - \frac{C_3 - 4rc}{C_1}}$$

However, since we also know that p must be between 0 and 1, we can bound rc from above and below to ensure that. Let us assume that $p = 0$; in this case, rc must be exactly $C_3/4 = \frac{3b_1+2b_2+b_3}{4} = A_1 - \bar{A}_0$. Let us now assume that $p = 1$; in this case, rc must be $\frac{C_1+C_2+C_3}{4} = \frac{b_1+2b_2+3b_3}{4} = A_4 - \bar{A}_3$. It can easily be shown that any rc between these two extremes will yield a solution for p between 0 and 1, and that there will be only one such solution because the other solution of the quadratic equation will fall outside the allowed region.

When we compare this case with case 5 (mmm) of the three-player case, we can see that the rc region is similar as it fills the space on the rc axis between the "everyone cheats" and the "everyone plays fair" region. However, a significant difference is that in the three-player case, the actual value of p was a linear function of rc, while in this case it depends on $\sqrt{rc}$. We conjecture that in the general n-player case, the value of p will depend on $(rc)^{1/(n-2)}$.

Finally, we can evaluate what happens when the prize structure is linear. In this case, $C_1 = C_2 = 0$ (since all the b's are equal), so the equation is not quadratic any more; we obtain

$$\frac{3}{2}b = rc$$

4.2.2 Case 7: three players cheat with certainty, one plays a mixed strategy (DDDm)

Without loss of generality, assume that players 1, 2 and 3 cheat with certainty. The equations are:

$$\begin{aligned} (1+2p_4)C_1 + (2+p_4)C_2 &\geq 12rc - 3C_3 \\ C_1 + C_2 + C_3 &= 4rc \end{aligned}$$

The second equation gives us a condition that must hold irrespectively of the actual value of p_4; it dictates that $\frac{b_1+2b_2+3b_3}{4} = A_4 - \bar{A}_3 = rc$. When this holds, the first equation is simplified to

$$(p_4 - 1)(2C_1 + C_2) \geq 0$$

and since $p_4 < 1$ (note that p_4 plays a mixed strategy; we have already treated the case of $p_4 = 1$ in case 1), it follows that we need $3b_3 - 2b_2 - b_1 \leq 0$. Therefore, this Nash equilibrium arises if the following two conditions both hold at the same time:

1. $rc = A_4 - \bar{A}_3$
2. $b_3 \leq \frac{2b_2+b_1}{3}$.

If both of the above conditions hold, any p_4 will satisfy the equations (including $p_4 = 0$ or $p_4 = 1$, which were already shown to be Nash-equilibria in Cases 1 and 2). Since any p_4 is suitable, this strategy then becomes DDD$*$.

For convex prize functions ($b_1 \geq b_2 \geq b_3$), the second condition trivially holds and all we need is $rc = A_4 - \bar{A}_3$. For linear prize functions, rc becomes $\frac{3}{2}b$. For concave prize functions ($b_1 \leq b_2 \leq b_3$), the second condition will never hold (unless $b_1 = b_2 = b_3$) and this case will not be a Nash-equilibrium.

4.2.3 Case 8: two players cheat with certainty, one plays fair with certainty, one plays a mixed strategy (DDFm)

Without loss of generality, assume that players 1 and 2 cheat with certainty, and player 3 plays fair. The equations are:

$$\begin{aligned} p_4C_1 + (1+p_4)C_2 &\geq 12rc - 3C_3 \\ (1+2p_4)C_1 + (2+p_4)C_2 &\leq 12rc - 3C_3 \\ C_1 + 2C_2 &= 12rc - 3C_3 \end{aligned}$$

Again, the third equation will give us a necessary condition for this configuration to be a Nash-equilibrium: $rc = A_3 - \bar{A}_2$. When this holds, the remaining two equations are as follows:

$$\begin{aligned} (p_4 - 1)(C_1 + C_2) &\geq 0 \\ p_4(2C_1 + C_2) &\leq 0 \end{aligned}$$

The implications are that $C_1 + C_2 \leq 0$ and $2C_1 + C_2 \leq 0$. Substituting C_1 and C_2 back yields the following two conditions for this Nash-equilibrium, both of which must be satisfied:

1. $rc = A_3 - \bar{A}_2$
2. $b_3 \leq \min(b_1; \frac{2b_2+b_1}{3})$.

If the above conditions both hold, any p_4 will satisfy the equations, including $p_4 = 0$ or $p_4 = 1$, which were already treated in Cases 2 and 3. Since any p_4 is suitable, this strategy then becomes DDF$*$.

For convex prize functions ($b_1 \geq b_2 \geq b_3$), the second condition trivially holds and all we need is $rc = A_3 - \bar{A}_2$. For linear prize functions, rc becomes $\frac{3}{2}b$. For concave prize functions ($b_1 \leq b_2 \leq b_3$), the second condition will never hold (unless $b_1 = b_2 = b_3$) and this case will not be a Nash-equilibrium.

4.2.4 Case 9: one player cheats with certainty, two play fair with certainty, one plays a mixed strategy (DFFm)

Without loss of generality, assume that player 1 cheats with certainty, and players 2 and 3 play fair. The equations are:

$$\begin{aligned} p_4C_2 &\geq 12rc - 3C_3 \\ p_4C_1 + (1+p_4)C_2 &\leq 12rc - 3C_3 \\ C_2 &= 12rc - 3C_3 \end{aligned}$$

Again, the third equation will give us a necessary condition for this configuration to be a Nash-equilibrium: $rc = A_2 - \bar{A}_1$. When this holds, the remaining two equations are as follows:

$$\begin{aligned} p_4 C_2 &\geq C_2 \\ p_4(C_1 + C_2) &\leq 0 \end{aligned}$$

The first equation implies that $C_2 \leq 0$ (since otherwise p_4 would have to be larger than 1, which is impossible). This is equivalent to $b_3 + 2b_2 \leq 3b_1$. The second equation implies that $C_1 + C_2 \leq 0$ (again, otherwise p_4 would have to be negative, which is impossible). This is equivalent to $b_3 \leq b_1$. Therefore, this Nash equilibrium arises if the following two conditions hold:

1. $rc = A_2 - \bar{A}_1$
2. $b_1 \geq \max(b_3; \frac{2b_2+b_3}{3})$.

If the above conditions both hold, any p_4 will satisfy the equations, including $p_4 = 0$ or $p_4 = 1$, which were already treated in Cases 3 and 4. Since any p_4 is suitable, this strategy then becomes DFF$*$.

For convex prize functions ($b_1 \geq b_2 \geq b_3$), the second condition trivially holds and all we need is $rc = A_2 - \bar{A}_1$. For linear prize functions, rc becomes $\frac{3}{2}b$. For concave prize functions ($b_1 \leq b_2 \leq b_3$), the second condition will never hold (unless $b_1 = b_2 = b_3$) and this case will not be a Nash-equilibrium.

4.2.5 Case 10: three players play fair with certainty, one plays a mixed strategy (FFFm)

Without loss of generality, assume that players 1, 2 and 3 play fair with certainty. The equations are:

$$\begin{aligned} p_4 C_2 &\leq 12rc - 3C_3 \\ 0 &= 12rc - 3C_3 \end{aligned}$$

The second equation says that this is a Nash-equilibrium only if $rc = \frac{3b_1+2b_2+b_3}{4} = A_1 - \bar{A}_0$. The first equation dictates that in this case, C_2 must be non-positive (because p_2 is non-negative and the right hand side is zero). Therefore, this Nash equilibrium arises if the following two conditions hold:

1. $rc = A_1 - \bar{A}_0$
2. $b_1 \geq \frac{2b_2+b_3}{3}$.

If both the above conditions hold, any p_4 will satisfy the equations, including $p_4 = 0$ or $p_4 = 1$, which were already treated in Cases 4 and 5. Since any p_4 is suitable, this strategy then becomes FFF$*$.

For convex prize functions ($b_1 \geq b_2 \geq b_3$), the second condition trivially holds and all we need is $rc = A_1 - \bar{A}_0$. For linear prize functions, rc becomes $\frac{3}{2}b$. For concave prize functions ($b_1 \leq b_2 \leq b_3$), the second condition will never hold (unless $b_1 = b_2 = b_3$) and this case will not be a Nash-equilibrium.

4.2.6 Case 11: two players cheat with certainty, two play a mixed strategy (DDmm)

Without loss of generality, assume that players 1 and 2 cheat with certainty. The equations are:

$$
\begin{aligned}
(p_3 + p_4 + p_3p_4)C_1 + (1 + p_3 + p_4)C_2 &\geq 12rc - 3C_3 \\
(p_3 + p_4 + p_3p_4)C_1 + (1 + p_3 + p_4)C_2 &\geq 12rc - 3C_3 \\
(1 + 2p_4)C_1 + (2 + p_4)C_2 &= 12rc - 3C_3 \\
(1 + 2p_3)C_1 + (2 + p_3)C_2 &= 12rc - 3C_3
\end{aligned}
$$

Again, the last two equations are symmetric w.r.t. p_3 and p_4 so we can replace them with a common p. The first two equations collapse into one:

$$
\begin{aligned}
(2p + p^2)C_1 + (1 + 2p)C_2 &\geq 12rc - 3C_3 \\
(1 + 2p)C_1 + (2 + p)C_2 &= 12rc - 3C_3
\end{aligned}
$$

Let us work out the second equation first:

$$
\begin{aligned}
(1 + 2p)C_1 + (2 + p)C_2 &= 12rc - 3C_3 \\
(2C_1 + C_2)p + C_1 + 2C_2 &= 12rc - 3C_3 \\
p &= \frac{12rc - 3C_3 - 2C_2 - C_1}{2C_1 + C_2} \\
p &= \frac{12rc - 4b_1 - 8b_2 - 6b_3}{3b_3 - 2b_2 - b_1}
\end{aligned}
$$

In order for the first equation to be true, we also need:

$$
\begin{aligned}
(2p + p^2)C_1 + (1 + 2p)C_2 &\geq (1 + 2p)C_1 + (2 + p)C_2 \\
(p^2 - 1)C_1 + (p - 1)C_2 &\geq 0 \\
(p - 1)((p + 1)C_1 + C_2) &\geq 0 \\
(p + 1)C_1 + C_2 &\leq 0 \\
p(b_3 - 2b_2 + b_1) &\leq 2b_1 - 2b_3
\end{aligned}
$$

At this stage, we must distinguish between three sub-cases:

- If $b_3 > 2b_2 - b_1$, it follows that $p \leq \frac{2b_1 - 2b_3}{b_3 - 2b_2 + b_1}$.
- If $b_3 < 2b_2 - b_1$, it follows that $p \geq \frac{2b_1 - 2b_3}{b_3 - 2b_2 + b_1}$.
- If $b_3 = 2b_2 - b_1$, the entire left hand side is zero, so we need $b_3 \leq b_1$.

We also have to ensure that the calculated p is between 0 and 1. $p > 0$ is satisfied when

$$rc > A_3 - \bar{A}_2 \qquad \text{and} \qquad 3b_3 > 2b_2 + b_1$$

or

$$rc < A_3 - \bar{A}_2 \qquad \text{and} \qquad 3b_3 < 2b_2 + b_1$$

while $p < 1$ is satisfied when

$$rc < A_4 - \bar{A}_3 \qquad \text{and} \qquad 3b_3 > 2b_2 + b_1$$

or

$$rc > A_4 - \bar{A}_3 \qquad \text{and} \qquad 3b_3 < 2b_2 + b_1$$

Note that we do not consider $p = 0$ or $p = 1$ because they have already been treated in cases 1 and 3. To sum it all up, this Nash-equilibrium will arise if the following conditions all hold at the same time:

1. $p = \frac{12rc - 4b_1 - 8b_2 - 6b_3}{3b_3 - 2b_2 - b_1}$
2. $A_3 - \bar{A}_2 < rc < A_4 - \bar{A}_3$ and $3b_3 > 2b_2 + b_1$
   or $A_4 - \bar{A}_3 < rc < A_3 - \bar{A}_2$ and $3b_3 < 2b_2 + b_1$
3. $b_3 > 2b_2 - b_1$ and $p \leq \frac{2b_1 - 2b_3}{b_3 - 2b_2 + b_1}$
   or $b_3 < 2b_2 - b_1$ and $p \geq \frac{2b_1 - 2b_3}{b_3 - 2b_2 + b_1}$
   or $b_3 = 2b_2 - b_1$, in which case $p = \frac{6rc + b_1 - 10b_2}{2b_2 - 2b_1}$

4.2.7 Case 12: one player cheats with certainty, one plays fair with certainty, two play a mixed strategy (DFmm)

Without loss of generality, assume that player 1 cheats with certainty and player 2 plays fair with certainty. The equations are:

$$\begin{aligned}
p_3p_4C_1 + (p_3 + p_4)C_2 &\geq 12rc - 3C_3 \\
(p_3 + p_4 + p_3p_4)C_1 + (1 + p_3 + p_4)C_2 &\leq 12rc - 3C_3 \\
p_4C_1 + (1 + p_4)C_2 &= 12rc - 3C_3 \\
p_3C_1 + (1 + p_3)C_2 &= 12rc - 3C_3
\end{aligned}$$

Again, we can assume a common $p = p_3 = p_4$ because of the last two equations:

$$\begin{aligned}
C_1p^2 + 2C_2p &\geq 12rc - 3C_3 \\
C_1p^2 + 2(C_1 + C_2)p + C_2 &\leq 12rc - 3C_3 \\
(C_1 + C_2)p + C_2 &= 12rc - 3C_3
\end{aligned}$$

Let us work out the third equation first:

$$p = \frac{12rc - 6b_1 - 8b_2 - 4b_3}{2b_3 - 2b_1}$$

Since we know that $0 < p < 1$, this Nash-equilibrium can arise only if

$$A_2 - \bar{A}_1 < rc < A_3 - \bar{A}_2 \qquad \text{and} \qquad b_3 > b_1$$

or

$$A_3 - \bar{A}_2 < rc < A_2 - \bar{A}_1 \qquad \text{and} \qquad b_3 < b_1$$

Additional conditions will arise from the first two equations:

$$\begin{aligned} C_1p^2 + (C_2 - C_1)p - C_2 &\geq 0 \\ C_1p^2 + (C_1 + C_2)p &\leq 0 \end{aligned}$$

Let us start with the second equation:

$$\begin{aligned} p(C_1(p+1) + C_2) &\leq 0 \\ C_1p &\leq -C_1 - C_2 \\ p(b_3 - 2b_2 + b_1) &\leq 2b_1 - 2b_3 \end{aligned}$$

which holds if $b_3 + b_1 \geq 2b_2$ and $p \leq \frac{2b_1-2b_3}{b_3-2b_2+b_1}$, or if $b_3 + b_1 \leq 2b_2$ and $p \geq \frac{2b_1-2b_3}{b_3-2b_2+b_1}$. When $b_3 + b_1 = 2b_2$, the left hand side is zero and we are left with $b_1 \geq b_3$.

The other equation yields two roots:

$$\begin{aligned} p_{1,2} &= \frac{(C_1 - C_2) \pm \sqrt{C_2^2 + C_1^2 + 2C_1C_2}}{2C_1} = \frac{C_1 - C_2 \pm (C_1 + C_2)}{2C_1} \\ p_1 &= 1 \\ p_2 &= -\frac{C_2}{C_1} = \frac{3b_1 - 2b_2 - b_3}{b_3 - 2b_2 + b_1} \end{aligned}$$

Now, if $C_1 < 0$, the equation is satisfied if p is between p_1 and p_2, and if $C_1 > 0$, the equation is satisfied if p is less than $\min(p_1, p_2)$ or greater than $\max(p_1, p_2)$. Let's cover all the four cases:

- If $C_1 < 0$ and $C_2 < 0$, p_2 is negative, so every p between 0 and 1 satisfies the equation, including $p = \frac{12rc-6b_1-8b_2-4b_3}{2b_3-2b_1}$ that we already determined.
- If $C_1 < 0$ and $C_2 > 0$, p_2 is positive. If $C_2 < -C_1$, we have $p > -\frac{C_2}{C_1}$, otherwise there is no valid p which gives a solution.
- If $C_1 > 0$ and $C_2 < 0$, p_2 is positive. If $C_2 < -C_1$, we have $p < -\frac{C_2}{C_1}$, otherwise there is no valid p which gives a solution.
- If $C_1 > 0$ and $C_2 > 0$, p_2 is negative, which excludes any valid p between 0 and 1 from being a solution.

To sum it all up, this Nash-equilibrium will arise if the following conditions all hold at the same time:

1. $p = \frac{12rc-6b_1-8b_2-4b_3}{2b_3-2b_1}$
2. $A_2 - \bar{A}_1 < rc < A_3 - \bar{A}_2$ and $b_3 > b_1$
   or $A_3 - \bar{A}_2 < rc < A_2 - \bar{A}_1$ and $b_3 < b_1$
3. $b_3 > 2b_2 - b_1$ and $p < \frac{2b_1-2b_3}{b_3-2b_2+b_1}$
   or $b_3 < 2b_2 - b_1$ and $p > \frac{2b_1-2b_3}{b_3-2b_2+b_1}$
   or $b_3 = 2b_2 - b_1$, in which case $p = \frac{12rc-2b_1-16b_2}{4b_2-4b_1}$
4. $b_3 < \min(2b_2 - b_1; 3b_1 - 2b_2)$
   or $b_3 < \min(b_1; 2b_2 - b_1)$ and $p > \frac{3b_1-2b_2-b_3}{b_3-2b_2+b_1}$
   or $b_3 > 2b_2 - b_1$ and $b_3 < b_1$ and $p < \frac{3b_1-2b_2-b_3}{b_3-2b_2+b_1}$

4.2.8 Case 13: two players play fair with certainty, two play a mixed strategy (FFmm)

Without loss of generality, assume that players 1 and 2 play fair with certainty. The equations are:

$$\begin{aligned} p_3p_4C_1 + (p_3+p_4)C_2 &\leq 12rc - 3C_3 \\ p_3p_4C_1 + (p_3+p_4)C_2 &\leq 12rc - 3C_3 \\ p_4C_2 &= 12rc - 3C_3 \\ p_3C_2 &= 12rc - 3C_3 \end{aligned}$$

Again, the last two equations are symmetric w.r.t. p_3 and p_4 so we can replace them with a common p. The first two equations collapse into one:

$$\begin{aligned} p^2C_1 + 2pC_2 &\leq 12rc - 3C_3 \\ pC_2 &= 12rc - 3C_3 \end{aligned}$$

Let us work out the second equation first:

$$p = \frac{12rc - 9b_1 - 6b_2 - 3b_3}{b_3 + 2b_2 - 3b_1} \tag{S7}$$

In order for the first equation to be true, we also need:

$$\begin{aligned} C_1p^2 + C_2p &\leq 0 \\ p(C_1p + C_2) &\leq 0 \\ C_1p + C_2 &\leq 0 \\ p(b_3 - 2b_2 + b_1) &\leq 3b_1 - 2b_2 - b_3 \end{aligned}$$

At this stage, we must distinguish between three sub-cases:

- If $b_3 > 2b_2 - b_1$, it follows that $p \leq \frac{3b_1-2b_2-b_3}{b_3-2b_2+b_1}$.
- If $b_3 < 2b_2 - b_1$, it follows that $p \geq \frac{3b_1-2b_2-b_3}{b_3-2b_2+b_1}$.
- If $b_3 = 2b_2 - b_1$, the entire left hand side is zero, so we need $3b_1 \geq 2b_2 + b_3$.

We also have to ensure that the calculated p is between 0 and 1. $p > 0$ is satisfied when

$$rc > A_1 - \bar{A}_0 \qquad \text{and} \qquad b_3 + 2b_2 > 3b_1$$

or

$$rc < A_1 - \bar{A}_0 \qquad \text{and} \qquad b_3 + 2b_2 < 3b_1$$

$p < 1$ is satisfied when

$$rc < A_2 - \bar{A}_1 \qquad \text{and} \qquad b_3 + 2b_2 > 3b_1$$

or

$$rc > A_2 - \bar{A}_1 \qquad \text{and} \qquad b_3 + 2b_2 < 3b_1$$

Note that we do not consider $p = 0$ or $p = 1$ because they have already been treated in cases 3 and 5. To sum it all up, this Nash-equilibrium will arise if the following conditions all hold at the same time:

1. $p = \frac{12rc - 9b_1 - 6b_2 - 3b_3}{b_3 + 2b_2 - 3b_1}$

2. $A_1 - \bar{A}_0 < rc < A_2 - \bar{A}_1$ and $b_3 + 2b_2 > 3b_1$
   or $A_2 - \bar{A}_1 < rc < A_1 - \bar{A}_0$ and $b_3 + 2b_2 < 3b_1$

3. $b_3 > 2b_2 - b_1$ and $p \leq \frac{3b_1 - 2b_2 - b_3}{b_3 - 2b_2 + b_1}$
   or $b_3 < 2b_2 - b_1$ and $p \geq \frac{3b_1 - 2b_2 - b_3}{b_3 - 2b_2 + b_1}$
   or $b_3 = 2b_2 - b_1$, in which case $p = \frac{12rc - 6b_1 - 12b_2}{4b_2 - 4b_1}$

4.2.9 Case 14: one player cheats with certainty, others play a mixed strategy (Dmmm)

Without loss of generality, assume that player 1 cheats with certainty. The equations are:

$$\begin{aligned}
(p_2p_3 + p_2p_4 + p_3p_4)C_1 + (p_2 + p_3 + p_4)C_2 &\geq 12rc - 3C_3 \\
(p_3 + p_4 + p_3p_4)C_1 + (1 + p_3 + p_4)C_2 &= 12rc - 3C_3 \\
(p_2 + p_4 + p_2p_4)C_1 + (1 + p_2 + p_4)C_2 &= 12rc - 3C_3 \\
(p_2 + p_3 + p_2p_3)C_1 + (1 + p_2 + p_3)C_2 &= 12rc - 3C_3
\end{aligned}$$

Again, the last three equations are symmetric w.r.t. p_2, p_3 and p_4 so we can replace them with a common p:

$$\begin{aligned}
C_1p^2 + C_2p + C_3 - 4rc &\geq 0 \\
C_1p^2 + 2(C_1 + C_2)p + C_2 + 3C_3 - 12rc &= 0
\end{aligned}$$

Both quadratic equations have two roots; let us work out the roots of the second equation first:

$$p = -\frac{C_1 + C_2}{C_1} \pm \sqrt{\left(\frac{C_1 + C_2}{C_1}\right)^2 - \frac{C_2 + 3C_3 - 12rc}{C_1}}$$

Since p must lie beween 0 and 1, we can also use the second equation to bound rc from above and below. Let us first assume that $p = 0$; in this case, $rc = \frac{C_2 + 3C_3}{12} = A_2 - \bar{A}_1$. Similarly, when $p = 1$, rc becomes $\frac{C_1 + C_2 + C_3}{4} = A_4 - \bar{A}_3$. Therefore, it must be ensured that rc is between $A_2 - \bar{A}_1$ and $A_4 - \bar{A}_3$ in order for this Nash-equilibrium to arise.

The first equation also gives us some further necessary conditions on rc; the roots here are similar to the roots of Case 6 (note that the equation is the same):

$$p_{1,2} = \frac{-C_2 \pm \sqrt{C_2^2 - 4C_1C_3 + 16C_1rc}}{2C_1} = -\frac{C_2}{2C_1} \pm \sqrt{\left(\frac{C_2}{2C_1}\right)^2 - \frac{C_3 - 4rc}{C_1}}$$

When $C_1 < 0$, p must be between p_1 and p_2; when $C_1 > 0$, p must be less than $\min(p_1, p_2)$ or greater than $\max(p_1, p_2)$.

4.2.10 Case 15: one player plays fair with certainty, others play a mixed strategy (Fmmm)

Without loss of generality, assume that player 1 plays fair with certainty. The equations are:

$$
\begin{aligned}
(p_2p_3+p_2p_4+p_3p_4)C_1+(p_2+p_3+p_4)C_2 &\leq 12rc-3C_3\\
p_3p_4C_1+(p_3+p_4)C_2 &= 12rc-3C_3\\
p_2p_4C_1+(p_2+p_4)C_2 &= 12rc-3C_3\\
p_2p_3C_1+(p_2+p_3)C_2 &= 12rc-3C_3
\end{aligned}
$$

Again, the last three equations are symmetric w.r.t. p_2, p_3 and p_4 so we can replace them with a common p:

$$
\begin{aligned}
C_1p^2+C_2p+C_3-4rc &\leq 0\\
C_1p^2+2C_2p+3C_3-12rc &= 0
\end{aligned}
$$

Both quadratic equations have two roots; let us work out the roots of the second equation first:

$$
p=-\frac{C_2}{C_1}\pm\sqrt{\left(\frac{C_2}{C_1}\right)^2-\frac{3C_3-12rc}{C_1}}
$$

Since p must lie beween 0 and 1, we can also use the second equation to bound rc from above and below. Let us first assume that $p=0$; in this case, $rc=\frac{C_3}{4}=A_1-\bar{A}_0$. Similarly, when $p=1$, rc becomes $\frac{C_1+2C_2+3C_3}{12}=A_3-\bar{A}_2$. Therefore, it must be ensured that rc is between $A_1-\bar{A}_0$ and $A_3-\bar{A}_2$ in order for this Nash-equilibrium to arise.

The first equation also gives us some further necessary conditions on rc; the roots here are similar to the roots of Case 6 (note that the equation is the same):

$$
p_{1,2}=\frac{-C_2\pm\sqrt{C_2^2-4C_1C_3+16C_1rc}}{2C_1}=-\frac{C_2}{2C_1}\pm\sqrt{\left(\frac{C_2}{2C_1}\right)^2-\frac{C_3-4rc}{C_1}}
$$

When $C_1>0$, p must be between p_1 and p_2; when $C_1<0$, p must be less than $\min(p_1,p_2)$ or greater than $\max(p_1,p_2)$.

4.3 Summary of the four-player case

All the conditions we have obtained for rc depended on four thresholds: $A_1-\bar{A}_0$, $A_2-\bar{A}_1$, $A_3-\bar{A}_2$ and $A_4-\bar{A}_3$. The behaviour of the model will primarily depend on the relation of rc to these thresholds as each region allows only specific types of Nash equilibria. Note that some of the cases as discussed above also depend on other criteria that are mostly related to the b_i variables that define the shape of the prize function.

In the three-player case that we discussed before, prize functions were categorized into three classes (concave, convex and linear ones) depending on the relation of b_1 to b_2. In the four-player case, we have b_1, b_2 and b_3, therefore the classification is not so straightforward. However, we can say that “realistic” prize functions are typically convex (i.e. $b_1\geq b_2\geq b_3$), and we can also evaluate linear ($b_1=b_2=b_3$) and concave ($b_1\leq b_2\leq b_3$) prize functions to complement our analysis and to make it comparable with the three-player case.

Let us start with the concave case:

rc	Cases of Nash-equilibria
Less than $A_4 - \bar{A}_3$	DDDD
Exactly $A_4 - \bar{A}_3$	DDDD, DDDF
Between $A_4 - \bar{A}_3$ and $A_3 - \bar{A}_2$	DDDF, DDmm, Dmmm, mmmm
Exactly $A_3 - \bar{A}_2$	DDDF, DDFF, Dmmm, mmmm
Between $A_3 - \bar{A}_2$ and $A_2 - \bar{A}_1$	DDFF, DFmm, Dmmm, Fmmm, mmmm
Exactly $A_2 - \bar{A}_1$	DDFF, DFFF, Fmmm, mmmm
Between $A_2 - \bar{A}_1$ and $A_1 - \bar{A}_0$	DFFF, FFmm, Fmmm, mmmm
Exactly $A_1 - \bar{A}_0$	DFFF, FFFF
Greater than $A_1 - \bar{A}_0$	FFFF

If the prize function is convex, the above table will look slightly different:

rc	Cases of Nash-equilibria
Less than $A_4 - \bar{A}_3$	DDDD
Exactly $A_4 - \bar{A}_3$	DDD$*$
Between $A_4 - \bar{A}_3$ and $A_3 - \bar{A}_2$	DDDF, DDmm, Dmmm, mmmm
Exactly $A_3 - \bar{A}_2$	DDF$*$, Dmmm, mmmm
Between $A_3 - \bar{A}_2$ and $A_2 - \bar{A}_1$	DDFF, DFmm, Dmmm, Fmmm, mmmm
Exactly $A_2 - \bar{A}_1$	DFF$*$, Fmmm, mmmm
Between $A_2 - \bar{A}_1$ and $A_1 - \bar{A}_0$	DFFF, FFmm, Fmmm, mmmm
Exactly $A_1 - \bar{A}_0$	FFF$*$
Greater than $A_1 - \bar{A}_0$	FFFF

For linear prize functions ($b = b_1 = b_2 = b_3$), the table is simplified to the following as all the thresholds collapse into a single one:

rc	Cases of Nash-equilibria
Less than $\frac{3}{2}b$	DDDD
Exactly $\frac{3}{2}b$	$****$
Greater than $\frac{3}{2}b$	FFFF

Finally, we present an alternative representation of the the different types of Nash equilibria on Figure S3. On this figure, each Nash equilibrium is represented by a box and Nash equilibria corresponding to the same rc value are arranged in the same column. The value of the rc product

increases from left to right. Arrows denote the "transition" of one type of Nash equilibrium into another (for instance, a DDDD type Nash equilibrium into Dmmm) as the value of rc changes. The upper panel contains the case of concave prize functions while the lower panel contains the convex case.

References

[1] Haugen KK (2004) The performance-enhancing drug game. Journal of Sports Economics 5: 67–86.

5 Figures

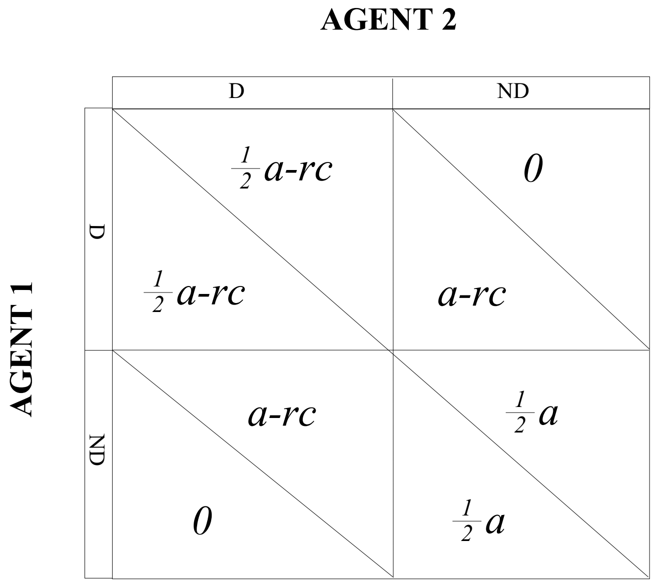

Figure S1: The original pay-off matrix from [1].

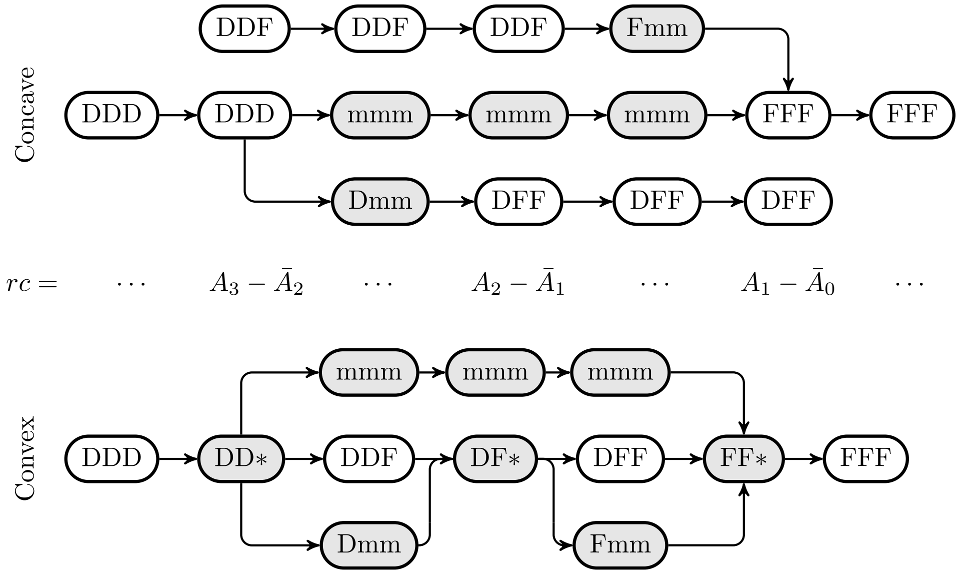

Figure S2: Nash equilibria in the three-player doping game as a function of the value of rc. Each Nash equilibrium is represented by a box. Nash equilibria with the same rc product are in the same column. rc increases from left to right. An arrow points from one Nash equilibrium into another if changing rc transforms a Nash equilibrium into another one.

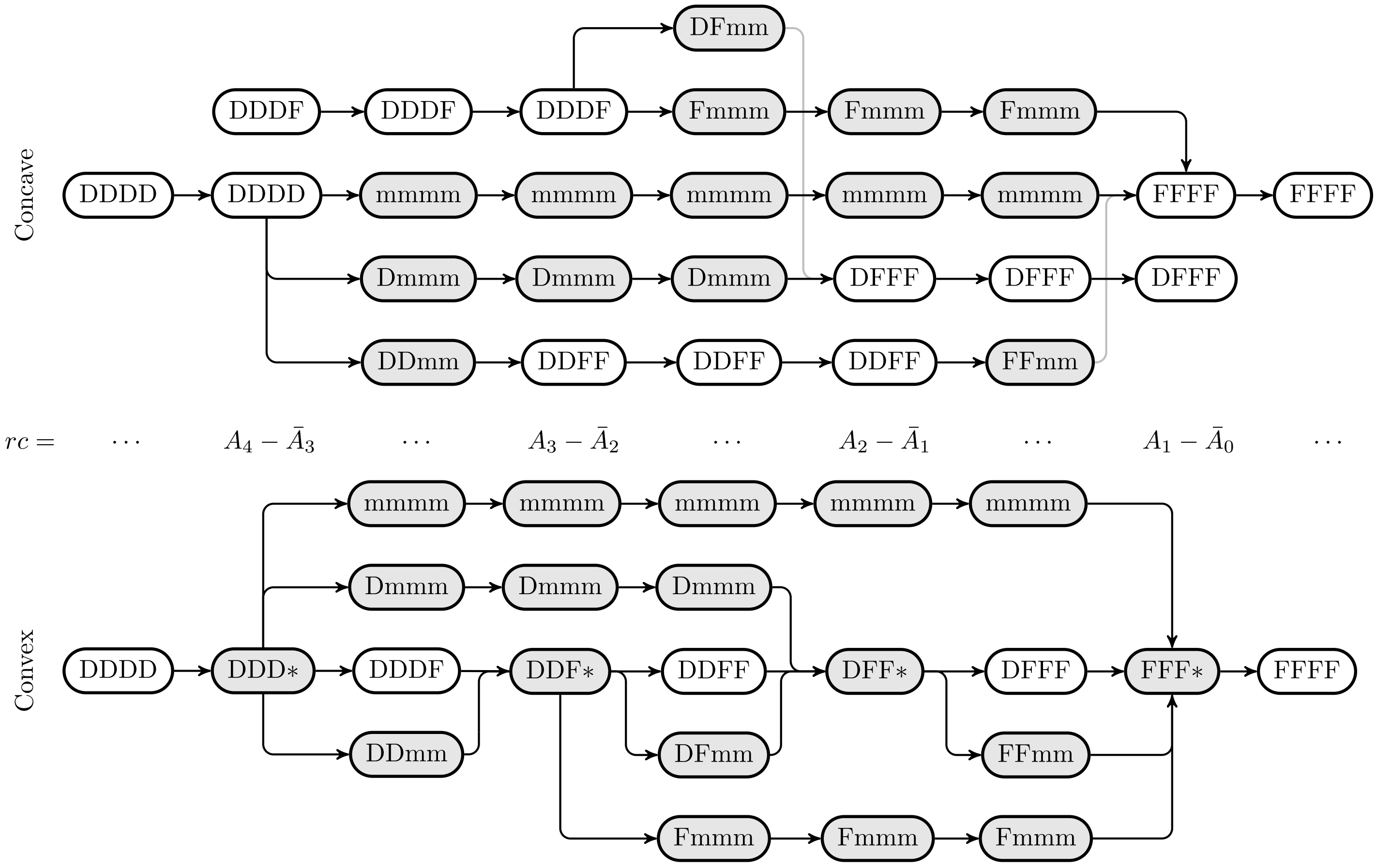

Figure S3: Nash equilibria in the four-player doping game as a function of the value of rc. Each Nash equilibrium is represented by a box. Nash equilibria with the same rc product are in the same column. rc increases from left to right. An arrow points from one Nash equilibrium into another if changing rc transforms a Nash equilibrium into another one.
